# A Novel and Powerful Dual-Stream Multi-Level Graph Convolution Network for Emotion Recognition

**DOI:** 10.3390/s24227377

**Published:** 2024-11-19

**Authors:** Guoqiang Hou, Qiwen Yu, Guang Chen, Fan Chen

**Affiliations:** 1 College of Intelligent Systems Science and Engineering, Harbin Engineering University, Harbin 150001, China; hgq15562858033@hrbeu.edu.cn (G.H.); yuqiwen@hrbeu.edu.cn (Q.Y.); 2 College of Intelligent Manufacturing, Chongqing Industry and Trade Polytechnic, Chongqing 401120, China; chenfan@cqgmy.edu.cn

**Keywords:** EEG, SEED, DEAP, emotion recognition, graph convolution network, multi-level graphs

## Abstract

Emotion recognition enables machines to more acutely perceive and understand users’ emotional states, thereby offering more personalized and natural interactive experiences. Given the regularity of the responses of brain activity to human cognitive processes, we propose a powerful and novel dual-stream multi-level graph convolution network (DMGCN) with the ability to capture the hierarchies of connectivity between cerebral cortex neurons and improve computational efficiency. This consists of a hierarchical dynamic geometric interaction neural network (HDGIL) and multi-level feature fusion classifier (M2FC). First, the HDGIL diversifies representations by learning emotion-related representations in multi-level graphs. Subsequently, M2FC integrates advantages from methods for early and late feature fusion and enables the addition of more details to final representations from EEG samples. We conducted extensive experiments to validate the superiority of our model over numerous state-of-the-art (SOTA) baselines in terms of classification accuracy, the efficiency of graph embedding and information propagation, achieving accuracies of 98.73%, 95.97%, 72.74% and 94.89% for our model as well as increases of up to 0.59%, 0.32%, 2.24% and 3.17% over baselines on the DEAP-Arousal, DEAP-Valence, DEAP and SEED datasets, respectively. Additionally, these experiments demonstrated the effectiveness of each module for emotion recognition tasks.

## 1. Introduction

Emotional expression is always accompanied by human behavior. This complexity poses a challenge for robots attempting to understand human needs, intentions and emotions. Effective human–robot interaction, therefore, requires robots to autonomously recognize emotional states from diverse sensory inputs. Emotion recognition technology has found applications across various fields, including healthcare, education, social media and autonomous driving. The types of sensory signals used for emotion recognition vary depending on the application. In education and autonomous driving, for example, facial expressions captured by cameras provide rich, visual, emotional information. In social media, interactions among users create an emotional corpus. In healthcare, the time–frequency features of physiological signals, e.g., ECG (electrocardiogram), EEG (electroencephalogram) and fMRI (functional magnetic resonance imaging), are closely linked to emotional states. However, both natural language and facial expressions are often influenced by human subjectivity, which can lead to misleading results. In contrast, physiological signals, due to their objectivity, demonstrate more objectivity in emotion recognition tasks. Among these, the EEG signal is garnering increasing interest from researchers due to its non-invasiveness and rich spatiotemporal information.

T.F. Bastos-Filho et al. embedded the average PSD (power spectral density) features across all EEG channels into a high-dimensional space and then took into account distance and label distribution to classify EEG using the KNN (K-nearest neighbor) algorithm [1]. The computational overhead of the distance between each sample and the others increases geometrically with the number of samples. G. Chanel et al. employed linear discriminant analysis (LDA) to refine the original features instead of mean pooling [2]. A nonlinear boundary between different classes of EEG in low-dimensional projection space is simply characterized by multiple linear hyperplanes. Y. Liu et al. constructed multiple linear decision hyperplanes with confidence intervals in the infinite-dimensional space using a support vector machine (SVM) with an RBF (radial basis function) kernel, achieving region partitioning of feature space [3]. The linear partition for a reproducing kernel Hilbert space (RKHS) largely depends on the efficiency of the artificial kernel function.

Considering the limitations of traditional machine learning methods such as SVM and LDA, researchers have focused on adaptive feature abstracters. Thammasan N. et al. employed pretrained deep belief networks (DBNs) to perform layer-wise representation learning for EEG and learn the relationship between emotion and EEG in the final layer [4]. Given that the model’s performance is sensitive to hyperparameters in a two-stage optimization task, deep learning has recently been incorporated in an end-to-end optimization strategy. Bhatti A.M. et al. combined a multilayer perceptron (MLP) with a BP neural network (NN) to learn the mapping from the original feature to final task-related representations in label space. The design of an MLP, the simplest architecture, lacks domain knowledge, and the no free lunch theorem [5] indicates that it is essential to design a dedicated architecture for a specific task. The spatiotemporal dynamics of multi-channel EEG signals encapsulate the intricate patterns of brain activity that correlate with various emotional states. Li J. et al. structuralized EEG signals to 3D feature maps, and convoluted a 2D feature map in each feature channel, which could capture the variation law of multi-channel signals [6]. The spatial information of an EEG contributes to emotion recognition. Compared to Li J., X. Li et al. considered the dependence on the sample sequence in capturing temporal patterns [7]. RNN is also used for learning spatially contextual information [8]. Both the RNN (recurrent neural network) and CNN (convolutional neural network) require the assumption of a relative position to structuralize the spatial distribution of channels. In practice, the spatial property of EEG signals only depends on the functional connectivity between brain electrodes. Without this assumption, channels and their functional connectivity can form an unstructured graph network.

The connectivity between nodes is regarded as the inductive bias of graph neural networks (GNNs) that are insensitive to isomorphic graphs. Graph convolution networks (GCNs) are used for layer-wise feature abstraction; then, combining the concatenation of the broad system with wide learning theory allows the model to search for features in a broad space. The functional connectivity of the EEG electrodes determines the spatial topology of the EEG channels but does not reflect the strength of the connections between channels. Tengfei Song et al. allowed for a trainable fraction adjacency matrix to explore the task-related correlation between channel signals [9]. Subcortical neurons may interact indirectly through deeper neurons, implying that the connectivity of cortical neurons is not static. Zhong P. et al. parameterized an adjacent matrix for dynamic connectivity and used the L2 regularization term for the sparsity of connectivity. The sensitivity of sense organs to an external stimulus varies between individuals, so the dynamics of a multi-channel EEG present some uncertainty. An instance-adaptive branch is responsible for instance-dependent features, and stochastic disturbance is introduced through a variational branch to enhance the model’s robustness [10]. Generally, the emotional state corresponds to a specific functional region of the brain. Instead of information fusion, Ye M. et al. extracted multi-level spatial information between EEG channels using a dual-stream GCN architecture to emphasize the regularity of brain activity [6]. Different from the feasible expression of adaptive connectivity, the introduction of expert knowledge can improve the interpretability of classification results and generalizability to specific tasks.

For the task of emotion recognition, a single feature or method for feature extraction is insufficient to learn representations in the label space. This motivates researchers to either consider more expert knowledge in architectural design or allow for more artificial features to be fed into networks. Currently, most researchers achieve this goal using dual-stream architectures, including DBGCN [11], GCB-BLS [12], V-IAG [10], HDGCN [6] and RGNN [13]. Sun M. et al. utilized two sequences of GNNs to extract emotion-related representations from PSD (power spectral density) and DE (differential entropy) features, respectively, thereby increasing the information capacity to enhance the upper limit of model performance [11]. Similarly, Zhang T. et al. enhanced the model’s own representation capacity through the wide learning theory and graph broad system [12]. Building on Zhang T. et al.’s work, Zhong P. et al. introduced a contrastive learning branch to more accurately learn emotion-invariant representations [13]. Given that human cognitive processes are largely identical but with minor differences, Song T. et al. enhanced the robustness of classifiers by introducing variational and subject-dependent branches, thereby improving the classification accuracy in subject-independent experiments [10]. Unlike previous work, Ye M. et al. recognized the regularity of the response of brain activity to human cognitive processes and proposed a dual-stream dynamic graph model for multi-level representation learning [6]. However, the connectivity between neurons exhibits hierarchical characteristics that GCNs in Euclidean space fail to capture. Additionally, layer-wise GCNs are required to identify the roles of nodes in the global scope, which is less efficient. To address the aforementioned issues, we separately introduce operators in hyperbolic space and random walk graph kernel tricks, constructing a powerful and novel dual-stream multi-level graph convolution network (DMGCN) for emotion recognition. The model includes a hierarchical dynamic geometric interaction neural network (HDGIL) and multi-level feature fusion classifier (M2FC). HDGIL is responsible for learning diverse representations across multi-metric spaces. Subsequently, M2FC is employed to make up for the forgotten details of EEG samples. The contributions of our study are as follows:

1. Novel architecture: We designed a powerful and novel dual-stream multi-level graph convolution network for emotion recognition with higher computational efficiency and diversified representations.
2. Benchmarking: We performed extensive experiments to compare the performance of the SOTA and our DMGCN against baselines, demonstrating the effectiveness of our module.

## 2. Related Work

### 2.1. Artificial Feature Extraction for EEG

Electroencephalogram acquisition devices can rapidly, synchronously sample a multi-channel EEG to obtain a high-resolution EEG sequence. The dynamics of an EEG gradually respond to a change in emotional state, so a clip of signals within sliding windows serves as an EEG sample. Generally, the signal-to-noise ratio (SNR) of an EEG increases with the sampling rate, but so does the computational overhead for signal processing and feature engineering, and the requirements for model performance become more exacting. Theoretically, a sampling frequency of 128 Hz corresponds to a Nyquist frequency of 64 Hz, which is sufficient for EEG-based emotion recognition tasks.

EEG signals, which are weak electrical signals emitted by cortical neurons, contain a large amount of information unrelated to emotional states and are also vulnerable to external interferences during acquisition, such as electromagnetic waves and skin sweat. Therefore, the preprocessing and feature engineering of EEGs are essential in the workflow. The preprocessing mainly involves removing power frequency artifacts in the environment with frequencies in the range of 50∼60 Hz, EOG (electrooculography) artifacts < 4 Hz caused by blinking, ECG artifacts around 1.2 Hz and EMG (electromyography) artifacts > 30 Hz. Techniques such as independent component analysis (ICA), discrete wavelet transform or bandpass filters (e.g., Butterworth bandpass filters) can be employed to eliminate noise in EEG data induced by external environmental factors. Additionally, further processing is required to remove various physiological artifacts. Artifact subspace reconstruction (ASR), as a method for enhancing artifact removal, utilizes a sliding window principal component analysis (PCA) to statistically interpolate any high-variance signal components that exceed a threshold. To address the issue of EEG baseline drift, it is recommended to employ the common average reference (CAR) method, which calculates the average of all the electrodes and subtracts it from each sample of every electrode [14].

EEG signals are statistically non-stationary, with their properties in either the time or frequency domain varying in response to brain activity. Therefore, feature engineering for EEGs focuses on joint time–frequency analysis rather than single frequency/time-domain analysis. The objective of joint time–frequency analysis is to analyze the variation in frequency-domain properties for a clip of signals within a sliding window. The mainstream analysis methods for time–frequency domain transformation include short-time Fourier transform (STFT) [15,16], wavelet analysis [17,18], etc. Wavelet analysis is often regarded as a “sparse representation” and excels at capturing subtle local features in the time–frequency domain. Consequently, it is widely used for processing non-stationary neural signals, particularly EEGs [19]. Compared to the STFT, wavelet analysis can flexibly adjust the resolution of both the time and frequency domains. Neural signals are approximately stationary within sliding windows. Under these conditions, the STFT is computationally more effective and intuitively more explicable than wavelet analysis. In the time–frequency domain, power spectral density (PSD) and differential entropy (DE) are most commonly employed. The best way to divide the frequency spectrum depends on the physiological state and cognitive processes in the brain, generally including Delta (1–4 Hz), Theta (4–8 Hz), Alpha (8–13 Hz), Beta (13–30 Hz) and Gamma (>30 Hz). Subsequently, the average powers of specific frequency bands are calculated as features, yielding the power spectral density (PSD) for the corresponding frequency bands [20,21]. Differential entropy (DE), as a representative nonlinear feature, assumes that the frequency distribution of EEG signals across various channels within a sliding time window follows a normal distribution, making it a generalization of information entropy for continuous signals. DE can describe the irregularity and uncertainty of EEG signals, and is more relevant than PSD to emotional state [22]. In addition to DE, García-Martínez et al. mention other nonlinear dynamical features in the time–frequency domains of EEGs [23].

### 2.2. Random Walk Graph Neural Networks

Graph neural networks (GNNs) belong to deep learning architectures designed for analyzing graph data. GNNs have recently been dominated by the three-stage paradigm of the message propagation neural network (MPNN), successively involving message passing, message aggregation and feature updating [24]. The recursive call for GNNs can broaden the receptive field—namely, the neighborhood radius—embedding more contextual information. The goal of the recursive call for GNNs is to obtain the task-related stationary distribution of node features. However, node features can suffer from over-smoothing and over-squashing as the number of neighbors geometrically rises. Therefore, the number of stacked layers becomes a critical factor affecting the performance of GNNs when designing GNNs based on the MPNN architecture. Additionally, there are models that follow novel design paradigms and do not belong to the MPNN family. A typical example is the random walk graph neural network (RWGNN) [25]. The model aims to capture a task-related feature subgraph within a specified neighborhood with random walk graph kernels. The random walk kernels are used for computing the similarities between k-hop subgraphs and hidden graphs. The similarity vector is a graph-level readout representation, and it is continually optimized to intuitively find feature subgraphs that play a decisive role in graph classification. In GNNs, the aggregation of contextual information is a crucial way to obtain graph-level structure-aware representation. Instead, RWGNN immediately recognizes task-related subgraphs through graph kernel tricks, but the radius of its neighborhood depends on the step size of the random walk. Inspired by implicit layer theory, Giannis Nikolentzos et al. proposed a geometric random walk graph neural network (GRWNN) capable of recognizing feature subgraphs between *∞*-hop neighborhoods at constant computational complexity [26].

### 2.3. Hyperbolic Graph Neural Networks

In the graph-level classification task, the neighborhood information embedded into a node feature, including the topological structure and node feature distribution, geometrically increases through layer-wise GCNs, while the capacity of the Euclidean space rises at a polynomial rate as a dimension. For the highest number of exponential nodes, it is difficult to maintain the distance-preserving properties in Euclidean space in higher-dimensional spaces, resulting in the distortion of the embedding [27]. The geometric growth of neighbors is essentially derived from the tree-like hierarchical structures of the graph. In hyperbolic space, negative curvature leads to the geometric expansion of space capacity, which is why the separate embedding of vast nodes is still manageable in lower dimensions [28]. The development of hyperbolic GNNs has recently attracted much attention among researchers. Hyperbolic operators are designed to realize the same operations in the Euclidean space. The isomorphic hyperbolic spaces mainly include Lorentz, Kelin and Poincaré ball, uniformly known as the Riemannian manifold, where both Lorentz and Poincaré ball are prevalent [29]. The expression of hyperbolic GNNs also relies on hyperbolic operators, similar to operations in the Euclidean space [29]. Zhang et al. proposed the first hyperbolic GNN, called the hyperbolic graph neural network (HGNN). Specifically, the GCN is performed in Euclidean space, and the embeddings are subsequently projected back to the Riemannian manifold [30]. Similarly, Chami et al. replaced the GCN and GAT module; however, unlike traditional GAT, HGAT utilizes both the Euclidean and hyperbolic distance to evaluate the attention scores of neighbors [2]. The hyperbolic operation of the above models consists of projection transformation and Euclidean operators. However, these models fail to adequately leverage the advantages of hyperbolic spaces. Lou A. et al. addressed this limitation by designing fully hyperbolic operators [31]. Additionally, Riemannian BatchNorm was developed to realize a normalization operation in the Riemannian manifold [31].

## 3. Methodology

**Figure 1:**
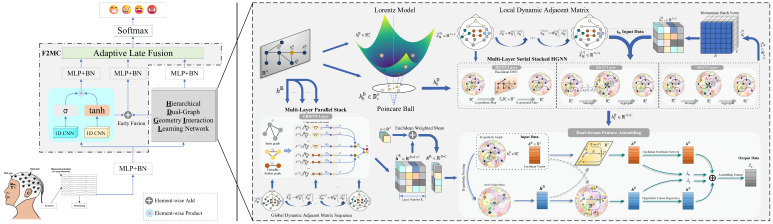
The architecture of DMGCN for EEG-based emotion recognition. DMGCN consists of construction of multi-level graph, hierarchical dynamic geometric interaction neural network (HDGIL) and multi-level feature fusion classifier (M2FC). We have an opportunity to focus on local and global connectivity of brain cortical neurons through construction of multi-level graph. HDGIL is a dual-stream model responsible for hierarchical graph representations and, finally, M2FC provides the method for adaptive fusion of these and classifying the graph.

In brain activities induced by emotions, the topological connectivity between EEG channels exhibits a hierarchical characteristic with dense intra-regional and sparse inter-regional connections [32]. The stereoscopic distribution of brain neurons ensures interaction between any neurons not subject to the arrangement of electrodes [6]. Given the hierarchical characteristics of brain activities and dynamic connectivity of cortical neurons, we propose the powerful and novel dual-stream multi-level graph convolution network (DMGCN) for emotion recognition, shown in Figure 1, which includes the hierarchical dynamic geometric interaction neural network (HDGIL) and multi-level feature fusion classifier (M2FC). In this section, we primarily focus on the implementation of the modules and provide detailed discussions on each module.

### 3.1. Construction of Multi-Level Graphs

The local graph is extracted from global graph through isolating functional connections between brain regions. EEG signals are regarded as a weighted yet undirected graph $G=\left(V,E,A\right)$, where the feature of *i*th node is $X_{i}\in R^{d_{f}}$ and edge set is E. When (i,j)∉E, the element of adjacent matrix $A_{ij}$ is 0; otherwise, it is a trainable parameter. For global $G_{G}^{\left(0\right)}=\left(V_{G}^{\left(0\right)},E_{G}^{\left(0\right)},A_{G}^{\left(0\right)}\right)$ and local $G_{R}^{\left(0\right)}=\left(V_{R}^{\left(0\right)},E_{R}^{\left(0\right)},A_{R}^{\left(0\right)}\right)$ dynamic graphs, only the topological structure is different, and the features of nodes are processed in the same way.

Feature engineering consists of artificial feature extraction and data normalization. Three-stage data cleansing, including Independent Component Analysis (ICA), Butterworth bandpass filter and Common Average Reference (CAR) methods, aims to enhance the signal-to-noise ratio (SNR), laying the foundation for subsequent analysis and feature extraction. Specifically, ICA is applied to remove physiological artifacts. Subsequently, a Butterworth bandpass filter (0 Hz∼75 Hz) is used to eliminate high-frequency noise and low-frequency drift. Finally, the issue of baseline drift is addressed by the CAR method. We obtain the Fourier spectrum of signal clips through sliding a 1 s Hamming window and STFT. After data cleansing, the DEs across five frequency bands, including δ (0.5 Hz∼4 Hz), θ (4 Hz∼8 Hz), α (8 Hz∼14 Hz), β (14 Hz∼31 Hz) and γ (31 Hz∼49 Hz), is computed to quantify the temporal variation of EEG uncertainty under different frequency conditions. Following the assumption that a clip of EEG signals follows the Gaussian distribution $x_{k}∼N\left(\mu_{k},\sigma_{k}^{2}\right)$, the DEs are formulated as follows:

(1) $$\begin{array}{cc} H\left(X_{k}\right) & =-\int_{a}^{b}\frac{1}{\sqrt{2\pi \sigma_{k}^{2}}}\exp\left(-\frac{{\left(x_{k}-\mu_{k}\right)}^{2}}{2\sigma_{k}^{2}}\right)\log\left(\frac{1}{2\pi \sigma_{k}^{2}}\exp\left(-\frac{{\left(x_{k}-\mu_{k}\right)}^{2}}{2\sigma_{k}^{2}}\right)\right)dx\\ \; & =\frac{1}{2}\log\left(2\pi e\sigma_{k}^{2}\right) \end{array}$$

In Equation (1), [a,b] is a Hamming window, and the index of frequency band k∈{δ,θ,α,β,γ}. The mean and variance of a clip of EEG signals is formulated as $\mu_{k}=\frac{1}{N}\sum_{j=1}^{N}x_{k}^{j}$ and $\sigma_{k}^{2}=\frac{1}{N}\sum_{j=1}^{N}{\left(x_{k}^{j}-\mu_{k}\right)}^{2}$, respectively, where $\left\{x_{k}^{1},x_{k}^{2},\cdots ,x_{k}^{S}\right\}$ is a sequence of discrete signals via sampling. To avoid gradient vanishing and accelerate convergence, normalization is performed for each channel of EEG signals.

(2) $${\tilde{X}}_{k}^{j}=\frac{X_{k}^{j}-\frac{1}{f}\sum_{j=1}^{f}X_{k}^{j}}{\sqrt{\frac{1}{f}\sum_{j=1}^{f}{\left(X_{k}^{j}-\frac{1}{f}\sum_{i=1}^{f}X_{k}^{j}\right)}^{2}}}$$

In Equation (2), the index of EEG channel is *j* and the number of frequency bands/the dimension of EEG channel f≤5. If not otherwise specified, the features of the node mentioned below refer to the concatenation of DEs of all frequency bands. Since the information received by the electrodes comes from all neurons in the local region of cerebral cortex, the correlation of adjacent channel signals exists. Therefore, singular value decomposition (SVD) is performed to eliminate redundant information.

(3) $$\hat{X}=V^{T}\tilde{X}$$

In Equation (3), $\left\{\hat{X},\tilde{X}\right\}\in R^{N\times f}$ represent the feature of nodes before and after SVD operation, respectively. The number of nodes is *N*, and the right singular matrix $V\in R^{N\times N}$. Initializing the weight of the adjacent matrix $A^{\prime }$ through Gaussian kernel function,

(4) $$\begin{array}{cc} A_{i,j}^{\prime } & =\left\{\begin{array}{cc} \exp\left(-\frac{1}{2}{∥X^{i}-X^{j}∥}_{2}^{2}\right)\; & A_{i,j}=1\\ 0\; & A_{i,j}=0 \end{array}\right.\\ \hat{A} & =D^{-\frac{1}{2}}A^{\prime }D^{-\frac{1}{2}} \end{array}$$

In Equation (4), *A* represents topological connectivity of electrodes, and $A^{\prime }$ is fractional adjacent matrix where $A_{i,j}^{\prime }$ indicates correlation between features of *i*th and *j*th nodes. The degree matrix $D_{i,i}=\sum_{j}A_{i,j}^{\prime }$ is a diagonal matrix whose diagonals are filled with the number of a node’s neighbors. $\hat{A}$ is a normalized adjacent matrix. Construction of multi-level graphs, including global and local graphs, is formulated as follows:

(5) $$A_{R}^{\left(0\right)}=\hat{A}⊙M_{R}\;A_{G}^{\left(0\right)}=\hat{A}\;X_{G}^{\left(0\right)}=X_{R}^{\left(0\right)}=\hat{X}$$

**Figure 2:**
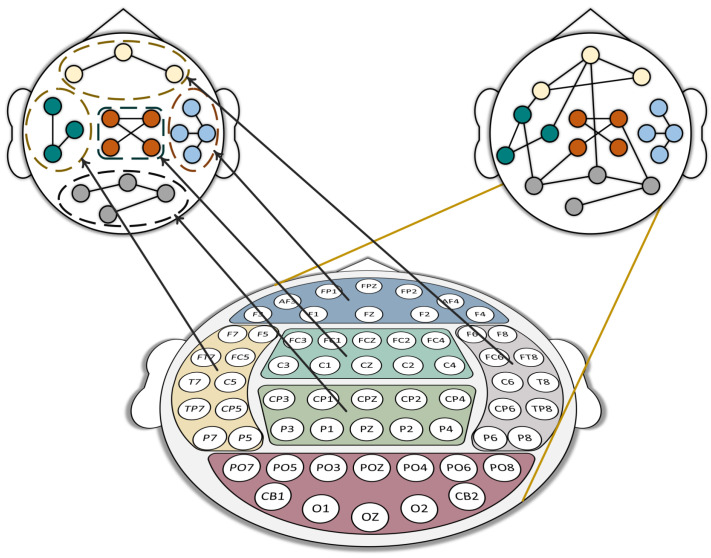
Construction of multi-level graphs.

In Equation (5), ⊙ is the element-wise product. $A_{R,G}^{\left(0\right)}\in R^{N\times N}$ and $X_{R,G}^{\left(0\right)}\in R^{N\times f}$ represent local or global topological structure and node feature, respectively. Masked matrix $M_{R}\in R^{N\times N}$ is used for isolation of brain regions, as shown in Figure 2. The arrangement of EEG electrodes fails to really reflect the dependence of cerebral cortical neurons. To address this issue, the connectivity between all electrodes that may mutually influence is parameterized, which adaptively learns dependencies between nodes at different levels of abstraction. The common method for updating global or local adjacent matrix is formulated as follows:

(6) $$\begin{array}{cc} {\bar{A}}^{\left(l\right)} & =\left(\sigma \left(W^{\left(l-1\right)}\right)⊙M\right)\left(A^{\left(l-1\right)}\right)\\ A^{\left(l\right)} & ={\left(D^{l}\right)}^{-\frac{1}{2}}{\bar{A}}^{\left(l\right)}{\left(D^{l}\right)}^{-\frac{1}{2}} \end{array}$$

In Equation (6), $\left\{A^{\left(l\right)},{\bar{A}}^{\left(l\right)}\right\}\in R^{N\times N}$ are separately the *l*th unnormalized and normalized adjacent matrix. The mask matrix *M* is $M_{R}$ if $A^{\left(l\right)}$ is the *l*th global adjacent matrix; otherwise, it is identity matrix $I_{N}$. The activate function $\sigma \left(\cdot \right)$ is used for element-wise nonlinear transformation of linear transformation matrix $W^{\left(l-1\right)}\in R^{N\times N}$. The *l*th degree matrix of $D^{\left(l\right)}$ is formulated as $D^{\left(l\right)}=\sum_{j}{\bar{A}}_{i,j}^{\left(l\right)}$. The construction of multi-level graphs lays the foundation for the exploration of hierarchical brain activity. To respect the pattern of brain activities, we develop a dual-stream graph architecture to extract emotion-related feature representation.

### 3.2. Hierarchical Dynamic Geometric Interaction Neural Network (HDGIL)

Zheng et al. found that the connectivity of EEG channels exhibits a tree-like hierarchical structure and the interaction pattern of intra-regional density and inter-regional sparsity [33]. Further, YE et al. experimentally discovered that different emotional states induce cooperative responses in relevant brain regions [6]. These findings are incorporated into our model design as expert knowledge. Since the role of node varies with the level of abstraction, the corresponding adjacent matrix requires being parameterized layer-by-layer to consider the dynamic correlation between node neighborhoods at different scales. For each layer, there are the local hierarchy checker (LHC) and global subgraph checker (GSC). The LHC is responsible for capturing locally tree-like hierarchical structures, and the GSC is aware of interactions between brain regions from a global perspective. Subsequently, both hyperbolic and Euclidean representations are mutually enhanced and then adaptively fused through Geometry Interactive Layer (GIL). Additionally, forward feedback network $\mathrm{FFN}:R^{f}\to R^{c_{\mathrm{in}}}$ is used for expansion of expression capacity before HDGIL.

#### 3.2.1. Local Hierarchy Checker (LHC)

The core of local hierarchy checker (LHC) is a hyperbolic graph attention network (HGAT) responsible for representation learning in hyperbolic space. Through layer-wise graph convolutions in Riemannian manifold, the tree-like structure can be hierarchically embedded into hyperbolic space. Additionally, Riemannian BatchNorm is introduced to align distribution of covariables.

The HGAT Layer is a hyperbolic vision of GAT based on the message propagation neural network (MPNN) paradigm, with its underlying implementation relying on operators in Table 1 and Table 2. First, linear transformation is used for dimensional expansion to construct heads.

(7) $$\begin{array}{cc} {\tilde{X}}_{R}^{i} & =W_{e_{h}}^{⊗}\left(\prod_{R\to M_{c_{i}}^{n}}\left(X_{R}^{i-1}\right)\right){⊕}_{c_{i}}b_{e_{h}}\\ {\hat{X}}_{R}^{i} & =\mathrm{reshape}\left({\tilde{X}}_{R}^{i},\left(N,c_{1}\right),\left(N,h,c_{h}\right)\right) \end{array}$$

In Equation (7), $X_{R}^{i-1}\in R^{N\times c_{\mathrm{in}}}$ and ${\tilde{X}}_{R}^{i}\in R^{N\times c_{1}}$ represent nodes’ features after and before linear transformation, respectively. $W_{e_{h}}\in R^{N\times c_{1}\times c_{\mathrm{in}}}$ and $b_{e_{h}}\in R^{N}$ are the weight and bias of linear transformation, respectively. Through $\mathrm{reshape}(\cdot ,\dim_{i},\dim_{o})$ operation, the $c_{1}$-dimensional feature of nodes is reshaped to h heads and the dimension of each head is $c_{h}$. ${\tilde{X}}_{R}^{i}\in R^{N\times h\times c_{h}}$ is multi-head feature of nodes. The parameter $c_{i}\in \left(-1,0\right)$ is negative curvature of hyperbolic space. The three stages of MPNN, including message passing, message aggregation and feature update, can be realized with the basic operators in Table 1. The calculation of attention score in HGAT takes into account both hyperbolic and Euclidean distances between EEG channels.

(8) $$\alpha_{R}^{x,i}=\mathrm{softmax}\left({\left[\left(\log_{0}^{c_{i}}\left({\tilde{X}}_{R}^{x,i}\right)∥\log_{0}^{c_{i}}\left({\tilde{X}}_{R}^{y,i}\right)\right)W_{R}^{i}\times d_{M}^{c_{i}}\left({\tilde{X}}_{R}^{x,i},{\tilde{X}}_{R}^{y,i}\right)\right]}_{y\in N_{x}}\right)$$

In Equation (8), $\left\{{\tilde{X}}_{R}^{x,i},{\tilde{X}}_{R}^{y,i}\right\}\in R^{c_{i}}$ are the *x*th and *y*th channels of the *l*th-layer nodes’ feature. The vectors $W_{R}^{i}\in R^{c_{h}}$ are used for adaptive suppression of feature space. $N_{x}$ is a set of channels connecting to *x*th channel. softmax(·) is to transform scalar vectors to probability distribution $\alpha_{R}^{x,i}\in R^{|N_{x}|}$, which serves as weights of asymmetric aggregation. Mean pooling is a permutation-invariant aggregation operator that aims to embed neighborhood structure and node-domain information into node feature. Fréchet Mean, a generation of Euclidean mean pooling to hyperbolic space, is employed to aggregate the information within the feature of 1-hop EEG channels.

(9) $${\overset{⏜}{X}}_{R}^{x,i}=\mathrm{Fr}\overset{´}{e}\mathrm{chet}\;\mathrm{Mean}\left({\left[{\hat{X}}_{R}^{y,i}\right]}_{y\in N_{x}}\cdot \alpha_{R}^{x,i}\right)$$

In Equation (9), ${\overset{⏜}{X}}_{R}^{i}\in R^{N\times h\times c_{h}}$ is the multi-head feature of *i*th-layer EEG channels. The Fréchet Mean(·) operation is formulated as Algorithms 1 and 2 in the different hyperbolic spaces (Appendix E [31]). Additionally, the backpropagation for Fréchet Mean(·) is an optimization (Appendix F [31]). Inspired by the channel attention mechanism [34], the adaptive fusion of old and new EEG channel features is achieved by scaling factors. The update of EEG channel features is formulated as follows:

(10) $$\begin{array}{cc} {\bar{X}}_{R}^{\left(i\right)} & ={\mathrm{reduce}}_{\mathrm{mean}}\left(\exp_{X_{R}^{\left(i-1\right)}}^{c}\left({\hat{X}}_{R}^{\left(i\right)}\times \tanh\left(\beta_{i}\right),h\times c,c\right)\right)\\ X_{R}^{\left(i\right)} & =X_{R}^{\left(i-1\right)}+\lambda \times {\bar{X}}_{R}^{\left(i\right)} \end{array}$$

In Equation (10), the *i*th-layer scaling factor $\beta_{i}$ determines mixing ratio of old and new information, and $X_{R}^{i}\in R^{h\times c_{k}}$ is the output of *i*th-layer HGAT. The mean pooling ${\mathrm{reduce}}_{\mathrm{mean}}\left(\cdot \right)$ is used for reduce multiple subspaces, the same as the settings of multi-head attention [35]. λ∈R is a scaling factor to adaptively fuse features. The different insensitivity of individuals to external stimulus leads to discrete distribution of EEG features in high-dimensional manifold. Alignment of distributions contributes to better generalization performance of the model for emotion recognition. To achieve this goal, Riemannian BatchNorm follows closely behind HGAT layer. The implementation of Riemannian BatchNorm is formulated as Algorithm 3. Riemannian BatchNorm can reduce shifts in the distribution of covariates between HGAT layers. Meanwhile, if neighboring HGATs have same curvatures, HGATs will degenerate to Vanilla GCNs. To address this potential problem, inter-layer nonlinear transformation is performed.

(11) $$\sigma^{c_{i},c_{j}}\left(x\right)=\exp_{0}^{c_{j}}\left(\sigma \left(\log_{0}^{c_{i}}\left(x\right)\right)\right)$$

In Equation (11), the element-wise activation function σ(·) generally chooses ReLU, LeakyReLU and Sigmoid due to shrink properties in Riemannian manifold [27]. The workflow of local hierarchy checker is formulated as Algorithm 4. The emotional state triggers the interaction of neurons across brain regions [20]. The presence of emotion has an effect on the diffusion of neurotransmitters across brain regions, and further requirement of emotion regulation gives rise to collaborative response of these regions. Therefore, it is also important for emotion recognition to pay attention to global task-related feature subgraphs.
**Algorithm 1** Lorentz model—Fréchet mean algorithm**Input:** 
$x^{\left(1\right)},\cdots ,x^{\left(t\right)}\in M_{c}^{n}\subseteq R^{n}$ and weight $w_{1},\cdots ,w_{t}\in R^{+}$;**Output:** 
$y_{T}=\underset{\mu \in M}{\arg\min}\frac{1}{t}\sum_{l=1}^{t}d\left(x^{\left(l\right)},\mu \right)$  1:$y_{0}=x^{\left(1\right)}$  2:**Loop**  3:    $u_{k+1}=\sum_{l=1}^{t}\left(w_{l}\cdot \frac{2\arccos^{-1}\left(-|K\right|{\langle x^{\left(l\right)},y_{k}\rangle }_{L})}{-\left|K\right|{\langle x^{\left(l\right)},y_{k}\rangle }_{L}^{2}}\right)\cdot x^{\left(l\right)}$  4:    $y_{k+1}=\frac{u_{k+1}}{\sqrt{-|K|\langle u_{k+1},u_{k+1}\rangle }}$  5:    **if** k≥T or $|y_{k+1}-y_{k}|\leq \mathrm{atol}$ or $\left|\frac{y_{k+1}-y_{k}}{y_{k}}\right|\leq \mathrm{rtol}$ **then**  6:        **return** $y_{k+1}$  7:    **end if**  8:**End Loop**

**Algorithm 2** Poincaré model—Fréchet mean algorithm
**Input:** 
$x^{\left(1\right)},\cdots ,x^{\left(t\right)}\in M_{c}^{n}\subseteq R^{n}$ and weight $w_{1},\cdots ,w_{t}\in R^{+}$;**Output:** 


$y_{T}=\underset{\mu \in M}{\arg\min}\frac{1}{t}\sum_{l=1}^{t}d\left(x^{\left(l\right)},\mu \right)$

  1:

$y_{0}=x^{\left(1\right)}$

  2:

$\mathrm{Define}\;g\left(y\right)=\frac{2\arccos^{-1}\left(1+2y\right)}{\sqrt{y^{2}+y}}$

  3:
**Loop**
  4:    **for** l=0,1,⋯,t **do**  5:        $\alpha_{l}=w_{l}g\left(\frac{|K|\cdot ∥x^{\left(l\right)}-y_{k}{∥}^{2}}{(1-|K|\cdot ∥x^{\left(l\right)}{∥}^{2})(1-|K|\cdot ∥y_{k}{∥}^{2})}\right)\;\frac{1}{1-|K|\cdot ∥x^{\left(l\right)}{∥}^{2}}$  6:    **end for**  7:    $a=\sum_{l=1}^{t}\alpha_{l},b=\sum_{l=1}^{t}\alpha_{l}x^{\left(l\right)},c=\sum_{l=1}^{t}\alpha_{l}{∥x^{\left(l\right)}∥}^{2}$  8:    $y_{k+1}=\left(\frac{\left(a+c|K|\right)-\sqrt{{\left(a+c|K|\right)}^{2}-4|K|\cdot {∥b∥}^{2}}}{2|K|\cdot {∥b∥}^{2}}\right)b$  9:    **if** k≥T or $|y_{k+1}-y_{k}|\leq \mathrm{atol}$ or $\left|\frac{y_{k+1}-y_{k}}{y_{k}}\right|\leq \mathrm{rtol}$ **then**  10:        **return** $y_{k+1}$  11:    **end if**  12:
**End Loop**
  13:**return** $y_{T}$


**Algorithm 3** Riemannian Batch Normalization
**Input:** 
All EEG channels in *i*th batch $X^{i}=\left\{x_{1}^{i},\cdots ,x_{n}^{i}\right\}$, Running mean $\tilde{\mu }\in M$, Running variance ${\left(\tilde{\sigma }\right)}^{2}\in R$, Target mean $\mu^{\prime }\in M$, Target variance ${\left(\sigma^{\prime }\right)}^{2}\in R$, EMA’s momentum β, size of *i*th batch $b_{i}$**Output:** 
${\hat{X}}^{i}=\left\{{\hat{x}}_{1}^{i},\cdots ,{\hat{x}}_{n}^{i}\right\}$, Running mean $\tilde{\mu }\in M$, Running variance ${\left(\tilde{\sigma }\right)}^{2}$  1:μ= Fréchet mean$\left(X^{i}\right)$, where Fréchet mean(·) refers to Algorithms 1 and 2  2:

$\sigma =\frac{1}{n}\sum_{l=1}^{n}{\left(x_{1}^{i}-\mu \right)}^{2}$

  3:**if** stage is train **then**  4:    ${\hat{X}}^{i}={\mathrm{Exp}}_{\mu^{\prime }}^{c}\left(\frac{\sigma }{\sigma^{\prime }}PT_{\mu \to \mu^{\prime }}^{c}\left({\mathrm{Log}}_{\mu }^{c}\left(X^{i}\right)\right)\right)$  5:    $\tilde{\mu }={\mathrm{Exp}}_{\tilde{\mu }}^{c}\left(\left(1-\beta \right){\mathrm{Log}}_{\tilde{\mu }}^{c}\left(\mu \right)\right)$  6:    $\tilde{\sigma }$ = Fréchet mean$\left(\left[\tilde{\sigma },\sigma \right],\left[1-\beta ,\beta \right]\right)$  7:
**else**
  8:    ${\hat{X}}^{i}={\mathrm{Exp}}_{\mu^{\prime }}^{c}\left(\sqrt{\frac{b_{i}-1}{b_{i}}}\times \frac{\tilde{\sigma }}{\sigma^{\prime }}PT_{\tilde{\mu }\to \mu^{\prime }}\left({\mathrm{Log}}_{\tilde{\mu }}^{c}\left(X_{i}\right)\right)\right)$  9:
**end if**



**Algorithm 4** The *i*th layer of local hierarchy checker
**Input:** 
EEG Feature $X_{R}^{i}$ and local adjacent matrix $A_{R}^{i}$ of the *i*th layer, output curvature $c_{in}$ and input curvature $c_{out}$ of the i+1th layer**Output:** 
EEG Feature of the i+1th layer $X_{R}^{i+1}$  1:Create multi-head for each channel of EEG                 ▹ Equation (7)  2:Compute edge weight, namely, aggregation weight              ▹ Equation (8)  3:Mean pooling on the manifold              ▹ Equation (9), Algorithms 1 and 2  4:Align distribution of features using Riemannian Batch Normalization     ▹ Algorithm 3  5:Adaptive feature update with residual connection                   ▹ (10)  6:Adaptive transformation of feature space                  ▹ Equation (11)  7:**return** EEG Feature $X_{R}^{i+1}$


#### 3.2.2. Global Subgraph Checker (GSC)

The global subgraph checker consists of BatchNorm (BN) and graph random walk neural network (GRWNN). The GRWNN Layer explicitly constructs trainable feature subgraphs, and then utilizes graph kernel tricks to calculate the similarity between them and EEG within the *∞*-hops neighborhood. For simplicity, omit the index of layer for all variables.

(12) $$\begin{array}{cc} \; & G_{\times }=\left(V_{\times },E_{\times },A_{\times }\right),\mathrm{where},\\ \; & \left\{\begin{array}{cc} \; & V_{\times }=\left\{\left(v,v_{i}^{\prime }\right)\in V_{g}\times V_{i}^{\prime }\right\},|V_{G}|=b\times N\\ \; & E_{\times }=\left\{\left(\left(v_{G},v_{i}^{\prime }\right),\left(u_{G},u_{i}^{\prime }\right)\right)\in V_{\times }V_{\times }|\left(u_{G},v_{G}\right)\in E_{G},\left(u_{i}^{\prime },v_{i}^{\prime }\right)\in E_{i}^{\prime }\right\} \end{array}\right.\\ \; & \kappa^{\infty }\left(G,G_{i}^{\prime }\right)=\mathrm{vec}{\left(q_{\times }\right)}^{T}{\left(I_{N}-\lambda A_{\times }\right)}^{-1}\mathrm{vec}\left(p_{\times }\right)\\ \; & {\tilde{x}}_{G}=\sigma \left({\left[\kappa^{\infty }\left(G,G_{i}^{\prime }\right)\right]}_{i=1,\cdots ,K}W+b\right)\\ \; & D_{\times }=\mathrm{diag}\left(d_{i}⊗d_{j}^{\prime }\right),\mathrm{where},\left\{\begin{array}{cc} d_{i} & =\sum_{j}A_{ij}=1^{T}A\\ d_{j}^{\prime } & =\sum_{i}A_{ij}^{\prime }=1^{T}A \end{array}\right.\\ \; & p_{\times }=D_{\times }\left(\mathrm{softmax}\left({\hat{X}}_{G}W_{\mathrm{start}}+b_{\mathrm{start}}⊗\mathrm{softmax}\left({\hat{X}}_{G}^{\prime }W_{\mathrm{start}}^{\prime }+b_{\mathrm{start}}\right)\right)\right)\\ \; & q_{\times }=D_{\times }\left(\mathrm{softmax}\left({\hat{X}}_{G}W_{\mathrm{stop}}+b_{\mathrm{stop}}⊗\mathrm{softmax}\left({\hat{X}}_{G}^{\prime }W_{\mathrm{stop}}^{\prime }+b_{\mathrm{stop}}\right)\right)\right) \end{array}$$

In Equation (12), the feature of nodes is $X_{G}\in R^{N\times f}$, the linear transformation matrix $W_{G}\in R^{f\times c_{h}}$,$\left\{W_{\mathrm{start}}^{\prime },W_{\mathrm{stop}}^{\prime }\right\}\in R^{c_{h}\times 1}$, bias is $\left\{b_{\mathrm{start}}^{\prime },b_{\mathrm{stop}}^{\prime }\right\}\in R$ and the degree matrix is $D_{\times }$ of product graph $G_{\times }$. The product graph $G_{\times }=\left(V_{\times },E_{\times },A_{\times }\right)$ consists of node feature $V_{\times }$, edge set $E_{\times }$ and adjacent matrix $A_{\times }$. The kernel function $\kappa^{\infty }\left(\cdot \right)$ is to compute similarity between global and feature graphs. $p_{\times }$ and $q_{\times }$ represent restart and end probability of node during random walking, respectively. The graph-level representation ${\tilde{x}}_{G}\in R^{K}$ is K graph kernel values, where K is the number of feature subgraphs. For a batch of graphs, the output of GWRNN is formulated as ${\tilde{X}}_{G}\in R^{N\times K}$. In order to ensure the dimensional compatibility of GSC and LHC, K is set to $c_{h}$. The computational complexity of ${\left(I_{N}-\lambda A_{\times }\right)}^{-1}$ is $O\left({\left(|V_{G}\times V_{G}^{\prime }\right)}^{3}\right)$. If $I_{N}-\lambda A_{\times }$ is an ill-condition matrix, the operation of matrix inversion may be accompanied by numerical instability. The implicit layer is introduced to address the issue, as shown in Algorithm 5, and corresponding backpropagation can refer to Theorem 1 [26]. Similarly, a BN is performed behind GWRNN, namely, $X_{g}=\mathrm{BN}\left({\tilde{X}}_{g}\right)\in R^{N\times K}$. Because of different metrics in hyperbolic and Euclidean space, traditional methods for feature fusion, such as mean, max and concatenation, are not applicable for both global subgraph checker (GSC) and local hierarchy checker (LHC). Therefore, conformal invariance is the fundamental requirement of design feature fusion method.
**Algorithm 5** Forward of GWRNN Layer**Input:** 
Feature graphs $G^{\prime }=\left(G_{0}^{\prime },\cdots ,G_{i}^{\prime },\cdots ,G_{n}^{\prime }\right)$, EEG graph *G*, Max Iteration T,rtol,atol**Output:** 
$\kappa^{\infty }\left(G,G^{\prime }\right)=\left(\kappa^{\infty }\left(G,G_{0}^{\prime }\right),\cdots ,\kappa^{\infty }\left(G,G_{i}^{\prime }\right),\cdots ,\kappa^{\infty }\left(G,G_{n}^{\prime }\right)\right)$  1:$\mathrm{Define}\;G=\left(V_{G},E_{G},A_{G}\right),G_{k}^{\prime }=\left(V_{k}^{\prime },E_{k}^{\prime },A_{k}^{\prime }\right)$  2:**function**f($z_{t},G,G_{k}^{\prime }$)  3:    $s=\mathrm{vec}(\sigma \left(XX_{k}^{\prime }\right))$  4:    $z_{t+1}=q_{\times }+\lambda \left(ss^{T}⊙A_{\times }\right)z_{t}$  5:    $\mathrm{return}\;z_{t+1}$  6:**end function**  7:**function** Iteration solver($z_{0},T,G,G_{k}^{\prime },\mathrm{rtol},\mathrm{atol}$)  8:    **Loop**                                         ▹ Calculate Fixed Point  9:         $z_{t+1}=$**F**$\left(z_{t},G,G_{i}^{\prime }\right)$  10:        $\mathrm{error}=z_{t+1}-z_{t}$  11:        **if** t≥T or |error|≤rtol or $\left|\frac{\mathrm{error}}{z_{t}}\right|\leq \mathrm{rtol}$ **then**  12:           $\mathrm{return}\;z_{t+1}$                        ▹ Iterate until Convergence or Max Iteration  13:        **end if**  14:    **End Loop**  15:**end function**  16:**for all** $G_{0}^{\prime },\cdots ,G_{i}^{\prime },\cdots ,G_{n}^{\prime }$ **do**  17:    $z^{★}$ = **ITERATION SOLVER**($z_{0}=0,T,G_{i}^{\prime },G,\mathrm{rtol},\mathrm{atol}$)  18:    $\kappa^{\infty }\left(G,G_{k}^{\prime }\right)=p_{\times }^{T}z^{★}$                                ▹ Store for Output  19:**end for**  20:$\mathrm{return}\;\kappa^{\infty }\left(G,G_{0}^{\prime }\right),\cdots ,\kappa^{\infty }\left(G,G_{i}^{\prime }\right),\cdots ,\kappa^{\infty }\left(G,G_{n}^{\prime }\right)$

#### 3.2.3. Geometry Interactive Layer (GIL)

In the Geometry Interactive Layer (GIL), graph-level representations are mutually projected into both metric spaces. Each graph-level representation can be enhanced through distance-aware adaptive fusion. It is worth mentioning that after the feature fusion, the node embeddings not only integrate different geometric characteristics via interaction learning but also maintain properties and structures of original space.  

(13) $$\begin{array}{cc} x_{R} & =\mathrm{Fr}\overset{´}{e}\mathrm{chet}\;\mathrm{Mean}\left(X_{R}^{L}\right)\\ {\hat{x}}_{G} & =\left(d_{M}^{c}{\left(\prod_{R^{n}\to M_{c}^{n}}\left(\exp_{0}^{c}\left(\prod_{R\to T_{0}M_{c}^{n}}\left(x_{G}\right)\right)\right),x_{R}\right)}^{2}\times \beta_{M}\right){⊗}_{c}x_{G}\\ x_{R}^{\prime } & =\prod_{R^{n}\to M_{c}^{n}}\left(x_{R}{⊕}_{c}{\hat{x}}_{G}\right)\\ x_{G}^{\prime } & =\beta_{E}\times {∥\log_{0}^{c}\left(x_{R}\right)-x_{G}∥}_{2}^{2}\times \log_{0}^{c}\left(x_{R}\right)+x_{G} \end{array}$$

In Equation (13), the scaling factors are $\beta_{M}$ and $\beta_{E}$. Local graph-level representation $x_{R}\in R^{c_{h}}$ is obtained through mean pooling Fréchet Mean(·) of node-level feature $X_{R}^{L}\in R^{N\times c_{h}}$. $x_{G}^{\prime }$ and $x_{R}^{\prime }$ are globally and locally enhanced representation. ${\hat{x}}_{G}$ is the hyperbolic vision of global representation $x_{G}$. Subsequently, linear transformations are employed to map the enhanced local and global EEG graph embeddings into a specified dimension feature space. The outputs of GIL are obtained through the adaptive weighted summation.

(14) $$Z_{G}=\alpha_{R}{\bar{x}}_{R}^{\prime }W_{R}+\beta_{G}{\bar{x}}_{G}^{\prime }W_{G},\mathrm{where},\alpha_{R}^{2}+\beta_{G}^{2}=1$$

In Equation (14), $\left\{W_{R},W_{G}\right\}\in R^{c_{h}\times c_{h}}$, the scaling factors are $\left\{\alpha_{R},\beta_{G}\right\}\in R$ and the graph-level representation of hierarchical dynamic geometric interaction neural network (HDGIL) is $Z_{G}\in R^{c_{h}}$. As the network deepens, the feature representation gradually loses the details of EEG samples. To address this issue, a method for multi-level feature fusion is essential for more comprehensive graph-level representations.

### 3.3. Multi-Level Feature Fusion Classifier (M2FC)

The multi-level feature fusion classifier (M2FC) consists of the Low-Level Feature Filter (LF) and Adaptive Fusion Classifier (AF). The M2FC is an enhanced residual network [36], which replaces Low-Level Feature Filter with residual connection. The Low-Level Feature Filter (LF) is responsible for preliminary cleaning of EEG samples, while the Adaptive Fusion Classifier (AF) performs the early–late adaptive fusion of features.

#### 3.3.1. Low-Level Feature Filter (LF)

Given that EEG samples include redundant information irrelevant to emotions, we utilize a gated 1D convolution network to selectively filter out task-related components.

(15) $$Z_{C}=\tanh\left(\Theta_{1}★X+b_{1}\right)⊙\sigma \left(\Theta_{2}★X+b_{2}\right)$$

In Equation (15), $\left\{\Theta_{1},\Theta_{2}\right\}$ are two 1D convolution kernels with bias $\left\{b_{1},b_{2}\right\}\in R$, and $Z_{C}$ is low-level representation of EEG samples, whose dimension depends on the properties of 1D convolution kernel. We flatten the EEG sample $\hat{X}\in R^{N\times f}$ to the 1D vector *X*.

#### 3.3.2. Adaptive Fusion Classifier (AF)

According to the time of the feature fusion, the methods for feature fusion are categorized into the counterparts of early and late fusion. Through early fusion, final representations retain a number of details. The methods for late fusion are performed for multiple representations with the ability of independent decision. The Adaptive Fusion Classifier (AF) integrates methods for early and late fusion to identity emotional states.

(16) $$\begin{array}{cc} {\hat{Z}}_{C} & =Z_{C}W_{C}+b_{C},\mathrm{where},W_{C}\in R^{c_{0}\times c_{h}},b_{C}\in R\\ Z_{f} & ={\hat{Z}}_{C}+Z_{G}\\ z_{f},z_{c},z_{g} & =Z_{F}W_{F}+b_{F},Z_{C}W_{C}+b_{C},Z_{G}W_{G}+b_{G},\mathrm{where},W_{*}\in R^{c_{h}\times c_{e}},b_{*}\in R\\ z & =\varphi_{f}\times z_{f}+\varphi_{c}\times z_{c}+\varphi_{g}\times z_{g}\\ \varphi_{f},\varphi_{c},\varphi_{g} & =\mathrm{softmax}\left(\left[z_{f},z_{c},z_{g}\right]W+b\right),\mathrm{where},W\in R^{c_{e}\times c_{e}},b\in R \end{array}$$

In Equation (16), $c_{0}$ and $c_{h}$ are the dimensions of convoluted EEG samples and graph-level representation from HDGIL. $c_{e}$ is the number of classes of emotional states. $\left\{z_{f},z_{g}\right\}\in R^{c_{e}}$ are decision-level features. $z_{c}\in R^{c_{e}}$ are low-level features with abundant details. $\left\{\varphi_{f},\varphi_{c},\varphi_{g}\right\}\in R$ are scaling factors for adaptive fusion of features. $z\in R^{c_{e}}$ is final representation learned by the DMGCN in label space.

### 3.4. The End-to-End Optimization of HDGIL

To accelerate training, we consider the classification loss of decision-level features $\left\{z_{c},z_{g}\right\}\in R^{c_{e}}$ as regularizations. Meanwhile, the L2 regularization of adjacent matrix is to hold sparsity of the graph. The classification loss is evaluated by cross-entropy loss function. Further, the entropy regularization is to smooth the multi-class joint probability distribution. Total loss is adaptively fused [37,38].

(17) $$\begin{array}{cc} {\hat{Y}}_{c},{\hat{Y}}_{g},\hat{Y} & =\mathrm{softmax}\left(z_{c}\right),\mathrm{softmax}\left(z_{g}\right),\mathrm{softmax}\left(z\right)\\ L_{1} & =L_{\mathrm{adj}}+L_{\mathrm{entropy}}=\left({∥{\hat{A}}_{R}^{L}∥}_{2}^{2}+{∥{\hat{A}}_{G}^{L}∥}_{2}^{2}\right)-\frac{1}{N}\sum_{i=1}^{N}\sum_{j=1}^{c_{e}}{\hat{Y}}_{i,j}\log\left({\hat{Y}}_{i,j}+\epsilon \right),\mathrm{where},\epsilon >0\\ L_{g},L_{c},L_{0} & =\Phi \left(Y,{\hat{Y}}_{g}\right),\Phi \left(Y,{\hat{Y}}_{c}\right),\Phi \left(Y,\hat{Y}\right),\mathrm{where},L_{2}=L_{g}+L_{c}\\ L & =\sum_{i=0}^{2}\frac{1}{2\sigma_{i}^{2}}L_{i}+\log\left(1+\sigma_{i}^{2}\right) \end{array}$$

In Equation (17), Φ(·) is cross-entropy loss function; the regularization for $L_{th}$ adjacent matrix is $L_{\mathrm{adj}}$, where *L* is the number of layers of LHC or GSC; and the regularization for entropy is $L_{\mathrm{entropy}}$. $L_{g},L_{c},L_{0}$ are classification losses of decision-level representations. $\left\{\sigma_{i},i=0,1,2\right\}$ are trainable parameters to adjust ratio of losses. ${\hat{Y}}_{c},{\hat{Y}}_{g},\hat{Y}$ are multi-class joint probability distribution predicted by decision-level representations. The end-to-end optimization for HDGIL is formulated as Algorithm 6.
**Algorithm 6** HDGIL Training Procedure**Input:** 
EEG Feature X and adjacent matrix A, True emotion label y, Number of layer M, Training epoch N, Learning rate lr**Output:** 
Predictive emotion label $\hat{y}$  1:Compute differential entropy of all frequency bands within sliding hamming windows as $\hat{X}$, where $\hat{X}=X_{G}^{\left(0\right)}=X_{G}^{\left(0\right)}$                  ▹ Equations (1)–(3)  2:Initialize adjacent matrix $\hat{A}$ by Gaussian distance and A; then, create local and global adjacent matrices $A_{R}^{\left(0\right)}$ and $A_{G}^{\left(0\right)}$                  ▹ Equations (4)–(6)  3:**for** current epoch ≤N **do**  4:    **for** i≤M**do**                      ▹ Create multi-level graphs  5:        Transform $A_{R}^{i-1},A_{G}^{i-1}$ to $A_{R}^{i},A_{G}^{i}$                  ▹ Equation (6)  6:        $X_{R}^{\left(i+1\right)}=\mathrm{LHChecker}\left(X_{R}^{\left(i\right)},A_{R}^{\left(i\right)}\right)$        ▹ Equations (7)–(11), Algorithm 4  7:        $X_{G}^{\left(i+1\right)}=\mathrm{GSChecker}\left(X_{G}^{\left(i\right)},A_{G}^{\left(i\right)}\right)$               ▹ Equation (12)  8:        $Z_{G}^{i+1}=\mathrm{GILayer}\left(X_{R}^{i},X_{G}^{i},A_{R}^{i}\right)$           ▹ Equations (13) and (14)  9:    **end for**  10:    $z,L_{reg}=M2\mathrm{FC}\left(Z_{G}^{M},X,y\right)$             ▹ Equations (15) and (16)  11:    $\hat{y}=\arg\max\left(\mathrm{softmax}(z)\right)$              ▹ Predict emotion label  12:    Compute $L=L_{cls}\left(\hat{y},y\right)+L_{reg}+L_{adj}+L_{diff}$          ▹ Equation (17)  13:    Carry out backpropagation algorithm for total loss L and update the learnable parameter Θ by Adam(lr=$\mathrm{lr},\beta_{1}=0.9,\beta_{2}=0.999$)  14:**end for**  15:return Predictive emotion label $\hat{y}$

## 4. Numerical Experiments

### 4.1. Emotional EEG Datasets

**SEED Dataset** [20,22]. Fifteen Chinese students, comprising seven males and eight females, served as subjects in the study. The mean and variance of their ages were 23.27 and 2.37, respectively. To protect privacy, their identifiers were replaced with numbers ranging from 1 to 15. Each subject participated in three experiments held at one-week intervals. To induce their emotions through visual and auditory stimuli, 15 four-minute film clips were elaborately selected from six movies as experimental materials, each accompanied by a 5-s prompt. During each experiment, subjects needed to sequentially watch these film clips, followed by a 45-s self-assessment and with a 15-s rest period after each viewing. Notably, the film clips were ordered to ensure consistency in emotional transformation. During viewing, EEG signals were collected using a 62-channel ESI NeuroScan System at a frequency of 1000 Hz, with the arrangement of electrodes following the 62-channel international “10–20” system standard. A sequence of EEG samples was labeled according to emotional state, as assessed by the subject. The SEED dataset provided preprocessed EEG samples produced through sequential down-sampling at 200 Hz, bandpass filtering from 0 to 75 Hz and feature engineering based on expert knowledge.

**DEAP Dataset** [14]. DEAP is a multimodal dataset designed for emotion classification tasks, including electrooculography (EOG), electromyography (EMG), the analysis of facial images, etc. Thirty-two physically and mentally healthy subjects, comprising sixteen males and sixteen females, were invited to participate in the experiment. The researchers sampled EEG signals at a frequency of 512 Hz using a 48-channel (32 EEG channels, 12 peripheral channels, three unused channels and one status channel) BIOSIG toolkit, and the data were collected using EEGLAB 14.1.2b for MATLAB R2024a.

The EEG electrodes were arranged according to the 32-channel international “10–20” system standard. The ratings from an online self-assessment for 120 one-minute extracts of music videos were each rated by volunteers based on arousal, valence and dominance. The data were recorded in two separate locations. Participants 1–22 were recorded in Twente and participants 23–32 in Geneva. Due to different revisions of the hardware, the order of EEG channels was different for the two locations. To ensure that the EEG samples were relevant to emotions, the data were segmented into 60-s trials, and a 3-s pre-trial baseline was removed. DEAP also provides preprocessed EEG samples produced through down-sampling at 128 Hz, bandpass filtering operations in the range of 4∼45 Hz, and feature engineering based on expert knowledge.

### 4.2. Experiment Settings

#### 4.2.1. Dataset Splitting

A method for K-fold cross-validations, to divide the training set and test set, was used to study the model’s performance for subject-independent experiments. Experiments were performed separately for each subject, where the data for all the trials for the subject were divided into K subsets in the trial dimension, with one subset being retained as the test set and the remaining K − 1 being used as training data. In our study, K was chosen to be the number of subjects (15 for SEED; 32 for DEAP). For the SEED dataset, we evaluated the model performance with sub-datasets from each experiment using the same method for data splitting, and the averages of metrics were used for comparison. Notably, the method for dataset splitting enabled us to intuitively evaluate the macro-accuracy of emotion recognition for each subject in subject-independent experiments.

#### 4.2.2. Baselines

The emerging dual-stream architecture has recently attracted researchers’ interests due to its ability for data augmentation, involving comprehensive feature extraction [11], robust representation learning [10,12,13] and the introduction of expert knowledge [6], including DBGCN [11], GCB-BLS [12], V-IAG [10], HDGCN [6] and RGNN [13]. In DBGCN [11], dual branches are responsible for the extraction of emotion-related representations from different kinds of EEG features to augment the correlation of the representation to emotions. GCB-BLS [12] improves the capacity of the representation space through enhancement nodes to obtain a robust classifier. Oppositely to the random projection of decision-level features, V-IAG [10] consists of an instance-adaptive and variational branch. The latter can alleviate the sparsity of the sample distribution in the representation space; therefore, the final classifier enables better generalization for unseen samples. In RGNN [13], one branch can approximate the optimal transportation between distributions of samples and emotion-invariant representations through contrastive learning; then, the combination of emotion-invariant with subject-dependent representations can suppress noise from the diversity of cognitive processes. Researchers of HDGCN [6] are aware of regular patterns of brain activities and have proposed the hierarchical architecture. Meanwhile, expert knowledge is introduced to improve the interpretability of the deep learning model to a certain extent. Comparisons with state-of-the-art baselines were made to highlight the superior embedding efficiency and competitive performance of our model.

#### 4.2.3. Experiment Environment

We implemented our work using Pytorch 2.2.0 [39] and employed the MPNN paradigm of the PyG library to design its convolution module [40]. The TorchEEG Library was used for data management, including preprocessing, random splitting and the construction of the data interface [41]. We performed end-to-end training on a multi-GPU system using Pytorch Lightning. In each experiment, the model was evaluated through K (15 for SEED; 32 for DEAP) cross-validations on two 12 GB NVIDIA GeForce RTX 3080Ti GPUs (NVIDIA Corporation, Santa Clara, CA, USA) and a 40-core Intel Xeon Gold 6248 CPU (Intel Corporation, Santa Clara, CA, USA).

#### 4.2.4. Experimental Protocol

In this experiment, we evaluated the performance of deep learning models in emotion recognition using the SEED and DEAP datasets, with the dataset-splitting methods and introduction outlined in Section 4.1 and Section 4.2.1, respectively. To assess the subject-independent emotion recognition capability of the models, we used leave-one-out cross-validation (LOOCV). This method, commonly used to evaluate model generalizability, is particularly effective for small datasets. In the subject-independent experiments, we aimed to evaluate the model’s ability to be generalized across the data manifolds of unseen individuals, testing its robustness to individual variations. Specifically, in each epoch, a part of the dataset from one subject serves as the test dataset, while the remaining datasets are used for training. This process is repeated until data from each subject have been used as the test dataset once. After LOOCV, statistics for the classification accuracy on test datasets are calculated. This avoids models learning individual-specific features and also reveals the model’s performance in handling individual variability.

#### 4.2.5. Hyperparameter Configuration

**Figure 3:**
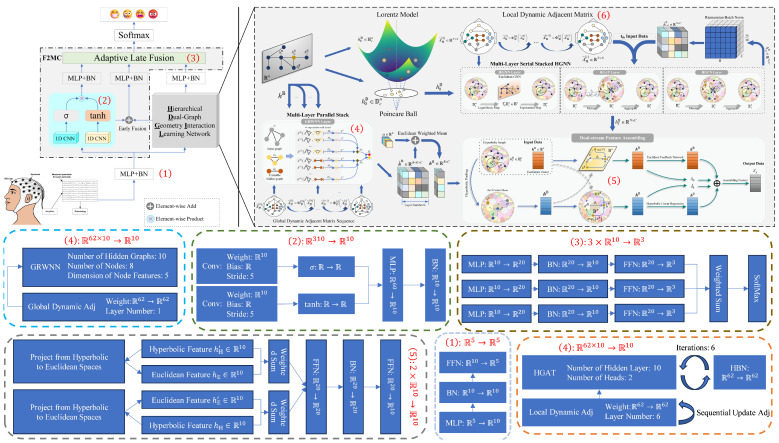
The module-level details of DMGCN. The weighted sum operator introduces parameters that allow an adaptive trade-off between different information, formulated as Z=α×X+(1−α)×Y, where $\alpha =\frac{\exp(\theta )}{1+\exp(\theta )}$ and θ∈R is a trainable parameter. HBN is short for the Riemannian BatchNorm layer to align the distributions of covariates in different layers. $F:X\times R^{a}\to R^{b}$ is a mapping from *X* vectors of dimension $R^{a}$ to a vector of dimension $R^{b}$, and $F:R^{a}\to R^{b}$ is regarded as one-to-one mapping, a special case where *X* is 1.

In our experiment, the hyperparameters were set as shown in Table 3 and Figure 3.

### 4.3. Experiment Results

#### 4.3.1. Recognition Accuracy

**Figure 4:**
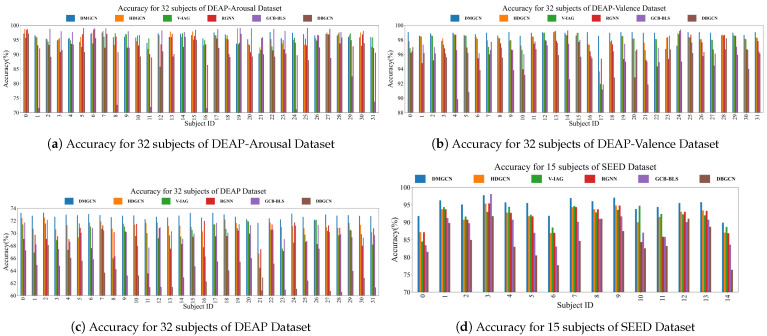
The visualization of classification accuracy for each subject in comparative experiments.

**Figure 5:**
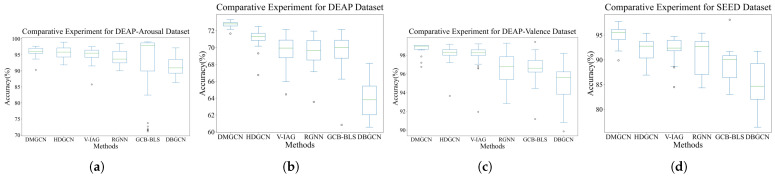
The visualization of models’ robustness in subject-independent comparative experiments. (**a**–**d**) are results from comparative experiment for DEAP-Arousal, DEAP, DEAP-Valence and SEED Datasets, respectively.

The results of comparative experiments are shown in Table 4 and Figure 4. The classification accuracy of DMGCN was improved by 0.59%, 0.32%, 2.24% and 3.17% compared to the SOTA baselines. Although DE features were more correlated with emotional states than PSD features, both types of features contained the same amount of information. This explains why incorporating more features did not yield the expected improvement in performance and resulted in the poorest performance of DBGCN. Among the baselines, HDGCN empirically demonstrates greater robustness in emotion recognition due to its prior knowledge being more aligned with physiologically objective regularity. Compared to the valence–arousal model in DEAP, the discrete emotion labels in SEED are more specific, making it easier for RGNN to extract emotion-invariant representations in the SEED dataset. Emotion states can be effectively represented in valence–arousal space. However, discretizing this two-dimensional space obscures the underlying relationships between emotional states and EEG samples, leading to a significant reduction in the accuracy of emotion recognition. The hierarchical characteristic with dense intra-regional and sparse inter-regional connections physiologically reflects the inherent patterns of cognitive processes in the human brain. Consequently, both DMGCN and HDGCN show more balanced classification performance for each subject through the exploration of intra- and inter-regional patterns that are specific to emotions, as shown in Figure 5. The higher efficiency of geometrical embeddings allows DMGCN to outperform HDGCN.

**Figure 6:**
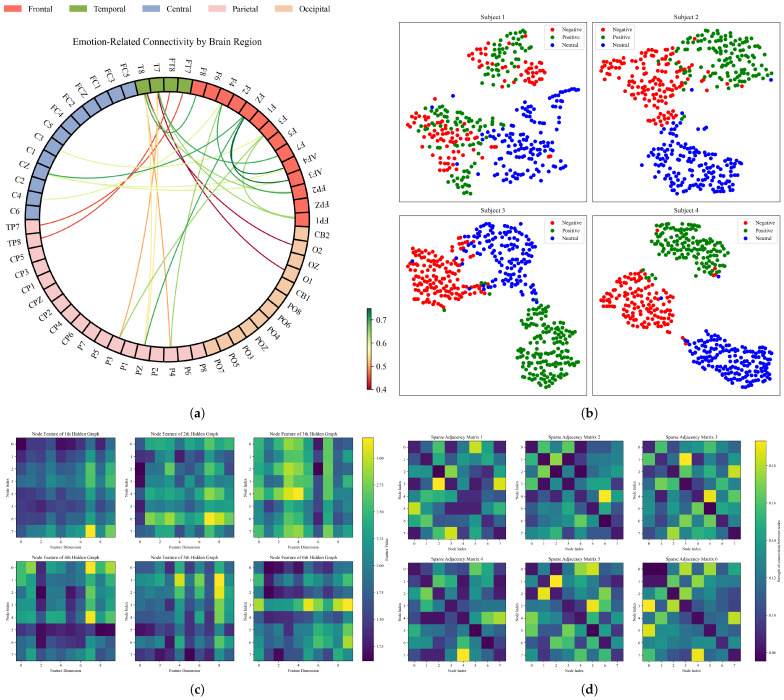
(**a**) For the SEED dataset, we visualize the top 24 connections with the highest strength. To prevent the values from being too close together and causing the colors to become indistinguishable, the weights of these connections are standardized. There is a symmetric connection strength between the nodes. (**b**) For the first four subjects of the SEED dataset, the output features of the M2FC were reduced using the t-SNE algorithm for visualization. (**c**) Visualization of the adjacent matrix of hidden graphs. (**d**) Visualization of the node features of hidden graphs.

Unlike DBGCN, which relies on a fully parameterized adjacency matrix, DMGCN transforms the original adjacency matrix layer by layer. This approach not only takes into account prior knowledge in the model design process but also enhances the flexibility of the representation. In Figure 6a,b, we visualize the global dynamic adjacency matrix from the final layer, and for the graph-level feature embeddings of the first four subjects, we applied t-SNE for dimensionality reduction and present the results in a 2D scatter plot. Additionally, Figure 6c,d show the hidden graph attributes, i.e., adjacency matrix and node features, within the GSC module, respectively.

#### 4.3.2. Embedding Efficiency

Research shows that neurons in the cerebral cortex are organized hierarchically, especially in sensory regions. This hierarchy means that neurons at different levels have distinct functional roles, facilitating the processing, integration and transmission of input information to higher levels, which contributes to complex cognitive and perceptual functions. This organization affects the electrical activity of neurons and is reflected in EEG signal characteristics. The negative curvature of hyperbolic GCNs is well-suited for modeling the hierarchy of neurons, offering significant advantages over Euclidean counterparts. Hyperbolic space naturally captures the hierarchical interactions of brain neurons, avoiding the over-squashing and over-smoothing issues common in vanilla layer-wise GCNs. To demonstrate the embedding efficiency resulting from negative curvature, we progressively reduced the dimensions of the hidden layers, decreasing their representation capacity, and compared the changes in parameter quantity with the corresponding accuracy in emotional recognition.

Because GSC and LHC are based on graph kernel tricks and constraint-preserved optimization, DMGCN can reduce memory usage at the cost of increased running time while enhancing the convexity of neural network optimization and minimizing redundant parameters. As shown in Table 5, DMGCN shows the smallest reduction in both parameter quantity and classification accuracy when the hidden-layer dimension decreases. Specifically, reducing the hidden-layer dimension by 2 results in average parameter reductions of 0.21 K, 1.00 K, 1.02 K, 0.91 K, 1.20 K and 0.50 K for DMGCN, HDGCN, RGNN, V-IAG, GCN-BLS and DBGCN, respectively. When the hidden-layer dimension is reduced to 4, the classification accuracy drops by 6.71%, 16.66%, 13.35%, 17.26%, 21.22% and 23.67% for DMGCN, HDGCN, RGNN, V-IAG, GCN-BLS and DBGCN, respectively. To achieve performance comparable to that of DMGCN, the hidden-layer dimensions of HDGCN, RGNN, V-IAG, GCN-BLS and DBGCN need to be increased to 6, 8, 8, 10 and 10, leading to additional parameter increases of 0.21 K, 0.92 K, 0.72 K, 0.73 K, 1.27 K and 0.34 K, respectively. This highlights that the negative curvature of hyperbolic GCNs effectively represents EEG’s hierarchy in lower dimensions with less information loss compared to Euclidean GCNs.

In GCN-BLS, feature-level fusion is applied to graph-level features from layer-wise GCNs, which are then fed into a broad learning system for random feature enhancement. This random enhancement network helps to guide the gradient flow of the classifier to avoid local optima during backpropagation. However, in the early training stages, when features are not strongly correlated with emotion categories, these random enhancement nodes may disturb the classifier’s gradient update direction. As more samples are observed, the gradient update direction becomes clearer. When the hidden-layer dimension decreases, the representation capacity of GCNs is limited, leading to more samples being utilized to create a robust classifier instead of enhancing feature effectiveness. Consequently, this reduction in hidden-layer dimension results in the most significant drop in classification accuracy for GCN-BLS in emotion recognition tasks. Among the baselines, RGNN’s performance is least affected by the hidden-layer dimension. RGNN uses a dual-branch architecture that combines contrastive learning with a series of GCNs to separately extract both subject-independent and subject-dependent features, allowing for certain sensitivity to important features even with limited representation capacity. However, because the feature extractor of the contrastive learning network is a sequence of GCNs, the representation capability of GCNs becomes a key bottleneck for improving RGNN’s performance. V-IAG enhances the classifier’s robustness to uncertainties, such as EEG noise and individual differences, through the variational branch. However, because each element in the variational adjacency matrix is a non-zero sampled value, feature updates require aggregating information from all nodes, which can lead to over-squeezing and over-smoothing, particularly when the hidden-layer dimension is lower. This is considered one reason for its decline in performance.

#### 4.3.3. Information Propagation

**Figure 7:**
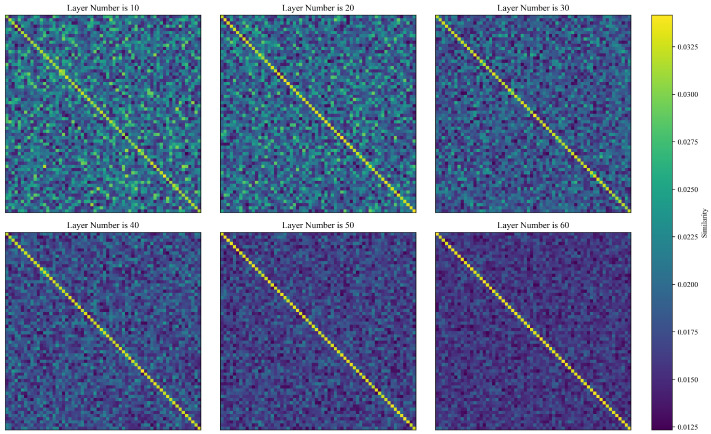
For example, in DBGCN [9], as GCN layers are stacked, node features gradually become less distinguishable for the SEED dataset. The similarity between node features is represented by the inner product of their feature vectors.

Topping et al. studied the effect of edge distribution on information propagation from the perspective of Ricci curvature [42]. As a descriptor of local geometric properties, the Ricci curvature reflects the density of the spatial connections among nodes. Specifically, when the Ricci curvature $r_{ij}>0$, the information flow between neighbor nodes i and j only goes through edge $e_{ij}$ between them. Conversely, when the Ricci curvature $r_{ij}>0$, the connectivity between neighbor nodes i and j is not limited by edge $e_{ij}$ between them. In regions of negative curvature, the information propagation between nodes may become blocked, leading to over-squashing. Given that the connectivity between brain neurons is characterized by “intra-region density and inter-region sparsity”, nodes that are connected across brain regions possess negative curvature and are located in discrete hyperbolic spaces. Vanilla GCNs are built on the message passing neural network (MPNN) paradigm, aiming to capture the geometric significance of nodes through layer-wise stacking to achieve a fixed point in representation space. However, in practice, layer-wise GCNs often suffer from over-squeezing. Meanwhile, frequency components propagate uniformly along the graph topology, resulting in excessively redundant information that overwhelms the useful information of the nodes themselves, resulting in over-squeezing being accompanied by over-smoothing (see Figure 7 for details). To enhance the graph embedding efficiency under sparse connectivity, DMGCN employs a geometric random walk neural network (GRWNN) as its GSC module. GRWNN, based on the random walk graph neural network (RWGNN) paradigm, approximates the effect of infinitely many GCN layers in a single step, significantly improving information propagation efficiency. To demonstrate DMGCN’s advantage in this regard over baselines, we examined the relationship between the number of GCN layers and the model’s classification performance.

All baselines utilize a dual-branch architecture, with each branch employing a sequence of GCNs for graph-level feature extraction. However, the layer-wise stacking of GCNs in a fixed-dimensional Euclidean space struggles to accommodate an exponential amount of information, leading to over-squeezing and over-smoothing. As shown in Table 6, in the case of the SEED dataset, the average classification accuracy of DBGCN, GCB-BLS, V-IAG and HDGCN begins to decline at layer numbers of 8, 10, 8 and 10, respectively. Among these, DBGCN, whose major parameters are used for its trainable adjacent, with its lower structural complexity, experiences the first performance drop around 12% when the layer number is 10. GCB-BLS, V-IAG and HDGCN also utilize sequences of GCNs, which causes their performance to deteriorate as the node feature discriminability decreases. Notably, HDGCN employs early–late adaptive fusion (AIM-AF), which helps to mitigate the lack of high-level abstract features by leveraging the details of raw data. Therefore, when the layer number is 10, the reduction in the classification accuracy of HDGCN is only 3.49%. In DMGCN, the GSC is a backpropagation-compatible GRWNN that integrates graph kernel tricks and implicit layer theory. With low feature dimensions, the combination of the pseudo-Newton method and the backpropagation framework allows for the rapid identification of the corresponding equilibrium point without the need for the layer-wise stacking of GCNs. This approach not only reduces the scale of the computational graph but also approximates the effect of infinitely many GCN layers in a single step. As shown in Table 6, the average classification accuracy of DMGCN levels off at layer number 10 and fluctuates slightly around 95% as the layer number increases. Notably, it is evident that DMGCN does not exhibit significant performance degradation. The GSC enables stable graph embeddings and prevents over-squashing during information propagation. Additionally, each HGAT in the LHC has varying curvatures, which helps the model to avoid degenerating into vanilla GCNs and mitigates the over-smoothing of node features. Moreover, the introduction of residual connections allows deep networks to approximate identity transformations, ensuring that model performance remains consistent even as the number of HGAT layers increases.

#### 4.3.4. Computation Complexity

Both the LHC and GSC modules in DMGCN are based on constraint-preserving optimization theory and feature closed-form mathematical formulations. Similar to physics-informed neural networks (PINNs), the introduction of well-defined models effectively guides parameter gradient flow, reducing the dependence on large labeled datasets and enabling rapid convergence. Moreover, the model emphasizes the inherent characteristics of physiological phenomena during training rather than merely fitting the training data. The integration of prior knowledge significantly enhances the model’s generalization capabilities, allowing it to perform effectively even with limited data. To empirically evaluate the computational complexity, i.e., the time and space complexity, we compared DMGCN with baselines in terms of floating-point operations (FLOPs), GPU memory usage (unit/Bytes) and computational density (FLOPs/Bytes).

Due to partial parameterization, both forward inference and backpropagation in DMGCN can be formulated as numerical iterative optimization problems. In computer systems, since the CPU handles numerical computations, DMGCN avoids excessive GPU memory usage during training. Unlike baselines that spend considerable time constructing computational graphs and performing error backpropagation, DMGCN allocates most of its FLOPs to numerical iterative optimization. Thus, the FLOPs of DMGCN remain roughly consistent across different datasets rather than relying heavily on parameter quantity. Although DMGCN and DBGCN have similar parameter counts, DMGCN’s time complexity is comparable to that of baselines. As shown in Table 7, on the SEED dataset, DMGCN shows a 35.6% increase in FLOPs and a 55% reduction in memory usage compared to the best baseline model, HDGCN. Meanwhile, it requires 5% more FLOPs for training DMGCN on the SEED dataset compared to DEAP-Arousal and DEAP-Valence. However, increased emotion categories may complicate root-finding on non-convex manifolds, potentially slowing model convergence. Overall, DMGCN outperforms baselines in terms of time and space complexity under the experimental setting.

### 4.4. Ablation Experiment

**Figure 8:**
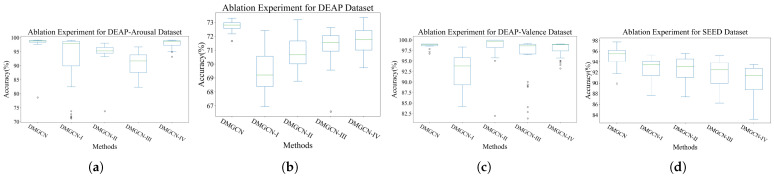
The visualization of models’ robustness in subject-independent ablation experiments. In the subfigures (**a**–**d**), we visualize the results of subject-independent ablation experiments in DEAP-Arousal, DEAP, DEAP-Valence and SEED Datasets. respectively.

**Figure 9:**
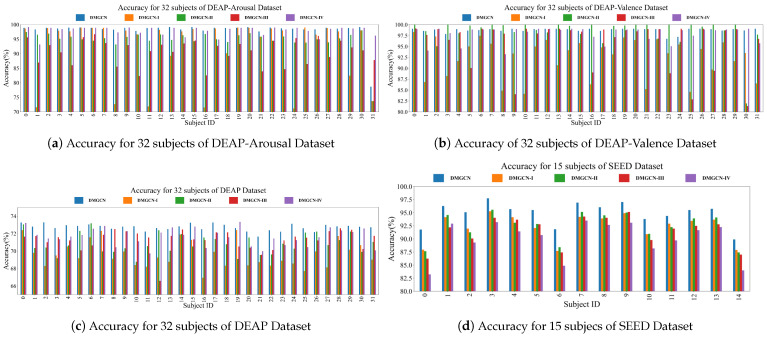
The visualization of classification accuracy for each subject in ablation experiments.

The results of the ablation experiments are shown in Table 8. The validity of each module was empirically verified in ablation experiments from the perspective of classification accuracy and subject-independent robustness, as shown in Figure 8 and Figure 9. The replacement of modules separately cause drops of up to 2.44%, 3.25%, 5.80% and 7.25% in classification macro-accuracy. Features in spaces whose metrics are different are immediately fed into the network, which violates conformal invariance. Meanwhile, because gradient descent and other optimization methods rely on consistent distance metrics, directly mixing features from different metric spaces may lead to slower convergence rates or even failure to converge to unified representations. Therefore, the replacement for GIL results in the greatest performance degradation. The replacement of M2FC with two-layer MLP has the least impact on the model’s performance, empirically suggesting that the emotion-related information mainly remains in the decision-level features. The key advantage of hyperbolic space over Euclidean space lies in its superior representation capacity, particularly in preserving hierarchical structures. This characteristic is a major factor behind LHC’s ability to improve model performance by up to 5.80%. Random diffusion of the node’s signal caused by isotropic aggregations in GCN raises the cost of information interaction between strongly connected nodes. This suggests that, in practice, layer-wise calls for the graph convolution operator fail to achieve the same effectiveness as the GSC. The characteristics of a single metric space may not be sufficient to capture all the necessary information. The GIL bridges the gap between spaces whose metrics are different from each other. Through geometrically mutual enhancement between features with different curvatures, the diversity of the information fed into classifiers can be enriched while maintaining conformal-invariance for each feature, thereby enhancing the classification performance.

**Table 8:** Average and variance of macro-accuracy in ablation experiment, where DMGCN-I∼DMGCN-IV are shown in Table 9. Bold indicates the highest average accuracy across all models, red highlights DMGCN’s performance, and the tilde shows the lowest variance during LOOCV.

Methods	DEAP-Valence		DEAP-Arousal		DEAP		SEED	
	ACC	STD	ACC	STD	ACC	STD	ACC	STD
DMGCN	**98.73**	1.15	**98.07**	10.25	**72.74**	0.82	**94.89**	3.93
DMGCN-I	98.41	9.01	97.93	2.97	71.66	1.81	92.45	4.08
DMGCN-II	97.90	2.92	94.82	12.17	71.29	3.02	92.34	3.78
DMGCN-III	95.87	8.91	92.27	13.94	70.86	2.21	91.59	4.46
DMGCN-IV	92.49	7.09	90.82	7.20	69.63	2.73	90.05	5.14

### 4.5. Sensitivity Analysis

#### 4.5.1. Hidden-Layer Dimension

As the dimensionality of the variables increases, constraint-preserved numerical optimization slows down or may even fail to converge. This is mainly due to the curse of dimensionality, which increases data sparsity, resulting in uniform distances between samples and ambiguous optimization directions. Furthermore, in high-dimensional spaces, the loss function becomes more complex, with a higher occurrence of local minima and saddle points, increasing the likelihood of falling into suboptimal solutions. To select an appropriate hidden-layer dimension, we evaluated the effect on model performance using the average and standard deviation of the classification accuracy, training time *T* and proportion ρ of samples that failed to converge within the maximum number of iterations in each epoch.

As shown in Table 10, when the hidden-layer dimension is set to 15, for the SEED dataset, more than 30% of samples fail to embed into reasonable positions within the maximum number of iterations and the classification accuracy begins to decrease. Increasing the hidden-layer dimension to 30 significantly prolongs the training time by doubling it. To reduce the risk of non-convergence during the iterative optimization process, we set the hidden-layer feature dimension to 10.

#### 4.5.2. Numerical Precision

During the optimization process, the settings for the absolute and relative errors directly affect the convergence precision and speed. A strict absolute error can trap the optimization in small local regions, slowing the convergence and risking overfitting. Conversely, a high relative error may prevent models from adequately responding to changes in the objective function, resulting in inaccurate results. Therefore, it is crucial to establish reasonable error settings that balance accuracy with convergence efficiency for effective optimization.

As shown in Table 11, setting the relative error to 1% often leads to the early termination of iterations, causing samples from different classes to occupy the same regions in the representation space. This overlap can reduce the model’s classification performance, resulting in lower average accuracy. With an absolute error of $10^{-5}$ and a relative error of 0.01, convergence within a limited number of iterations is difficult. As a result, approximately 70% of the samples reached the maximum iteration limit, leading to increased training time. It is clear that the dominance of absolute and relative errors varies by dataset. When the relative error is set to 0.1%, changes in absolute error led to a steady improvement in DMGCN’s classification accuracy on the SEED dataset, with no significant model degradation observed at an absolute error of $10^{-5}$. In contrast, changes in absolute error resulted in model degradation for DMGCN on the DEAP-Arousal/Valence dataset. With an absolute error of $10^{-4}$ and a relative error of 0.1%, DMGCN achieved a desirable classification stability, average training time and divergent ratio across most of the datasets. Therefore, in the experimental setup, we set the absolute and relative errors for numerical optimization to $10^{-4}$ and 0.1%, respectively.

## 5. Conclusions

Considering that the cognitive processes of humans influence the interaction patterns of neurons in the cerebral cortex, we designed a novel data-driven model for emotion recognition by incorporating expert knowledge. This model employs a dual-stream architecture to extract emotion-related representations in multi-level graphs and naturally enriches the diversity of feature representations through a conformal invariance-based method for the fusion of features with different curvatures. Additionally, early and late feature fusion compensates for the tendency of deep networks to forget details of the sample. Extensive experiments conducted on existing mainstream datasets (SEED and DEAP) validated that our model outperforms current state-of-the-art baselines in terms of embedding efficiency and classification accuracy, and empirically verified the functional effectiveness of each module. We have pioneered the application of hyperbolic graph deep learning models in emotion recognition tasks, but also observed issues with the optimization not converging as the feature space dimension increased. We hope that researchers can build upon our work to explore more stable iterative methods for optimization in hyperbolic space, thereby further enhancing the representation capacity of hyperbolic space.

## Figures and Tables

**Table 1:** The basic operators in the Poincaré ball and the Lorentz model (c<0). Hyperbolic manifolds are low-dimensional projections of higher-dimensional hyperbolic surfaces, with prevalent models being the Poincaré ball and the Lorentz model. Both possess advantages in intuitive visualization and numerical stability, respectively. Both $\exp_{x}^{c}\left(v\right)$ and $\log_{x}^{c}\left(v\right)$ are a pair of reciprocal mappings between manifold and tangent space. If ${∥v∥}_{2}^{2}\to 0,\exp_{x}^{c}\left(v\right)\approx x+v$; similarly, if ${∥x-v∥}_{2}^{2}\to 0,\log_{x}^{c}\left(v\right)\approx x-v$. Additionally, the metric is ${\langle x,y\rangle }_{L}=g_{x}^{L_{c}^{n}}\left(x,y\right)=-x_{0}y_{0}+x_{1}y_{1}+\cdots +x_{n}y_{n}$ in Riemannian manifold.

	Poincaré Ball	Lorentz
Manifold	$B_{c}^{n}:=\left\{x\in R^{n}:{∥x∥}_{2}<-\frac{1}{c}\right\}$	$L_{c}^{n}=\left\{x\in R^{n+1}:{\langle x,x\rangle }_{L}=-\frac{1}{c},x_{0}>0\right\}$
Tangent Space	$T_{x}B_{c}^{n}:=\left\{v\in R^{n+1},x\in B_{c}^{n}:{\langle v,x\rangle }_{L}=0\right\}$	$T_{x}L_{c}^{n}:=\left\{v\in R^{n+1},x\in L_{c}^{n}:{\langle v,x\rangle }_{L}=0\right\}$
Origin	$o_{B}=\left[0,0,\cdots ,0\right]\in R^{n}$	$o_{L}=\left[-1,0,\cdots ,0\right]\in R^{n+1}$
Metric	$g_{x}^{B_{c}^{n}}={\left(\lambda_{x}^{c}\right)}^{2}g^{E^{n}},\mathrm{where},\lambda_{x}^{c}=\frac{2}{1+{c∥x∥}_{2}^{2}},g^{E^{n}}=I_{n}$	$g_{x}^{L_{c}^{n}}=\eta$,where η is *I* expect $\eta_{0,0}=-1$
Induce Distance	$d_{B}^{c}\left(x,y\right)=\frac{1}{\sqrt{∥c∥}}\cosh^{-1}\left(1-\frac{{2c∥x-y∥}_{2}^{2}}{\left(1+{c∥x∥}_{2}^{2}\right)\left(1+{c∥y∥}_{2}^{2}\right)}\right)$	$d_{L}^{c}=\frac{1}{\sqrt{∥c∥}}\cosh^{-1}\left(c{\langle x,y\rangle }_{L}\right)$
Exponential Mapping	$\exp_{x}^{c}\left(v\right)=x{⊕}_{c}\left(\tanh\left(\sqrt{\left|c\right|}\frac{\lambda_{x}^{c}{∥v∥}_{2}}{2}\right)\frac{v}{\sqrt{{|c|∥v∥}_{2}}}\right)$	$\exp_{x}^{c}\left(v\right)=\cosh\left(\sqrt{\left|c\right|}{∥v∥}_{L}\right)x+v\frac{\sinh\left(\sqrt{\left|c\right|}{∥v∥}_{L}\right)}{\sqrt{{|c|∥v∥}_{L}}}$
Logarithmic Mapping	$\log_{x}^{c}\left(y\right)=\frac{2}{\left|c\right|\lambda_{x}^{c}}\tanh^{-1}\left(\sqrt{\left|c\right|}{∥-x{⊕}_{c}y∥}_{2}\right)\frac{-x{⊕}_{c}y}{∥-x{⊕}_{c}{y∥}_{2}}$	$\log_{x}^{k}\left(y\right)=d_{L}^{k}\left(x,y\right)\frac{y+\frac{1}{k}{\langle x,y\rangle }_{L}x}{{∥y+\frac{1}{k}{\langle x,y\rangle }_{L}x∥}_{L}}$
Parallel Transport	${\mathrm{PT}}_{x\to y}^{c}\left(y\right)=\frac{\lambda_{x}^{c}}{\lambda_{y}^{c}}\mathrm{gyr}\left[y,-x\right]v$	${\mathrm{PT}}_{x\to y}^{c}\left(v\right)=v-\frac{c{\langle x,y\rangle }_{L}}{{∥1+c{\langle x,y\rangle }_{L}∥}_{L}}\left(x+y\right)$
Scalar Multiplication	$r{⊗}_{c}x:=\frac{1}{\sqrt{\left|c\right|}}\tanh\left(r\tanh^{-1}\left(\sqrt{\left|c\right|}∥x∥\right)\right)\frac{x}{∥x∥}$	$r{⊗}_{c}x:=\exp_{0}^{c}\left(r\log_{0}^{c}\left(x\right)\right)$
Bias Addition	$x{⊕}_{c}y=\frac{\left(1+2c\langle x,y\rangle +{c∥y∥}_{2}^{2}\right)x+\left(1-{c∥x∥}_{2}^{2}\right)}{1+2c\langle x,y\rangle +c^{2}{∥x∥}_{2}^{2}{∥y∥}_{2}^{2}}$	$x{⊕}_{c}y=\exp_{x}^{c}\left({\mathrm{PT}}_{0\to x}^{c}\log_{0}^{c}\left(y\right)\right)$
Matrix-Vector Production	$M^{⊗}\left(x\right)=\tanh\left(\frac{∥Mx∥}{∥x∥}\arctan\left(∥x∥\right)\right)\frac{Mx}{∥Mx∥}$	$M^{⊗}\left(x\right)=\exp_{0}^{c}\left(M\log_{0}^{c}\left(x\right)\right)$
Mapping in Manifold	$\sigma^{c_{i},c_{j}}\left(x\right)=\exp_{0}^{c_{j}}\left(\sigma \left(\log_{0}^{c_{i}}\left(x\right)\right)\right),\sigma \in \left\{\mathrm{ReLU},\mathrm{LeakyReLU},\mathrm{Sigmoid}\right\}$	

**Table 2:** Projections between hyperbolic and Euclidean space (c<0).

	Poincaré Ball	Lorentz
Project from Euclidean E to Manifold M	$\prod_{R^{n}\to B_{c}^{n}}\left(x\right):=\frac{x}{\left(\sqrt{\left|c\right|}+\epsilon \right){∥x∥}_{2}},\mathrm{where},\epsilon >0$	$\prod_{R^{n+1}\to L_{c}^{n+1}}\left(x\right):=\left[\sqrt{\frac{1}{c}+{∥x_{1:n}∥}_{2}^{2}},x_{1},\cdots ,x_{n}\right]$
Project from Euclidean E to Tangent $T_{x}M$	$\prod_{R^{n}\to T_{x}B_{c}^{n}}:=x$	$\prod_{R^{n+1}\to T_{x}L_{c}^{n}\left(v\right)}:=v+c{\langle x,v\rangle }_{L}x$

**Table 3:** Summary of hyperparameters in our experiments. As the dimension of the hidden layer increases, convergence to the feasible solution becomes more difficult, so the dimension of the hidden layer is set to a lower value, namely, 10. Under these settings, the effect of the representation capacity of the space on the performance of the model can be visually observed.

Hyperparameter	Value
Learning Rate, $\left(\beta_{1},\beta_{2}\right)$ ^1^	$10^{-3},\left(0.9,0.999\right)$
Batch Size for SEED and DEAP	20
Dropout Rate ^2^	0.1
Initial Curvature for Riemannian Manifold ^3^	−0.5

^1^ Parameter was updated using the Adam optimizer. ^2^ Dropout operation applied only to the M2FC module. ^3^ The curvatures for *L* layers are trainable parameters with non-zero and negative initial values.

**Table 4:** Average and variance of the macro-accuracy in a comparative experiment. The labeling methods for DEAP-Valence, DEAP-Arousal and DEAP differ from one another. In DEAP-Valence and DEAP-Arousal, sample classification is based on the relative position of valence and arousal values with respect to their thresholds (0.5) on the corresponding axes, respectively. In contrast, DEAP considers both valence and arousal to label samples, resulting in a label space with four dimensions. Valence represents the positivity/negativity levels of emotions, while arousal reflects the activation level of emotional states. Additionally, bold indicates the highest average accuracy across all models, red highlights DMGCN’s performance, underlining marks the best accuracy among baselines, and the tilde shows the lowest variance during LOOCV.

Methods	DEAP-Valence		DEAP-Arousal		DEAP		SEED	
	ACC	STD	ACC	STD	ACC	STD	ACC	STD
DBGCN	95.03	4.18	91.74	5.38	63.78	3.77	84.95	7.67
GCB-BLS	96.64	4.12	92.27	13.94	69.46	5.66	88.88	7.53
V-IAG	96.72	3.23	94.17	4.28	69.53	4.20	90.97	5.54
RGNN	97.94	3.63	94.96	5.88	69.65	3.85	91.97	5.12
HDGCN	98.16	2.76	95.67	3.55	71.14	2.88	91.74	4.22
DMGCN	**98.73**	1.15	**95.97**	3.68	**72.74**	0.82	**94.89**	3.93

**Table 5:** Effect of hidden-layer dimension on model performance. The model’s average classification accuracy (%) was evaluated using leave-one-out-cross-validation (LOOCV), while the standard deviation (%) of accuracy indicates the model’s robustness to individual differences. Additionally, Params refers to the number of floating-point parameters and its unit is K.

*D*	Models	SEED			DEAP			DEAP-Arousal			DEAP-Valence		
		ACC	STD	Params	ACC	STD	Params	ACC	STD	Params	ACC	STD	Params
4	DBGCN	61.28	7.93	5.0	43.75	4.93	5.1	70.23	8.66	5.1	69.41	2.46	5.1
	GCB-BLS	67.66	6.87	6.5	47.05	3.15	6.5	75.22	6.27	6.5	77.49	5.02	6.5
	V-IAG	73.71	4.66	9.3	54.14	4.81	9.5	77.93	4.72	9.5	78.19	4.56	9.5
	RGNN	78.62	3.99	10.3	55.63	2.71	10.3	75.34	3.70	10.3	80.52	3.80	10.3
	HDGCN	75.08	4.46	14.2	59.44	3.48	12.7	79.46	2.22	12.7	82.23	3.36	12.7
	DMGCN	88.18	3.75	6.4	67.60	2.36	5.6	87.31	4.95	5.6	89.07	2.11	5.6
6	DBGCN	72.01	5.95	5.2	47.97	6.71	5.4	75.72	5.36	5.4	74.71	3.79	5.4
	GCB-BLS	71.45	4.41	7.5	54.66	4.66	7.5	80.83	5.97	7.5	82.96	1.72	7.5
	V-IAG	79.99	3.50	10.2	56.75	3.29	10.4	83.95	3.60	10.4	83.43	5.60	10.4
	RGNN	80.66	3.04	11.2	59.52	5.69	11.2	80.34	4.70	11.2	83.52	3.49	11.2
	HDGCN	81.39	4.93	15.4	64.44	3.70	13.9	95.38	3.06	13.9	87.31	1.47	13.9
	DMGCN	89.24	2.85	6.9	69.95	4.25	6.2	89.01	4.32	6.2	91.54	3.02	6.2
8	DBGCN	77.85	6.34	5.5	59.69	4.11	5.6	83.72	4.95	5.6	85.93	5.65	5.6
	GCB-BLS	81.77	5.74	8.5	62.54	4.08	8.5	85.74	4.97	8.5	87.82	3.31	8.5
	V-IAG	82.10	4.67	11.1	64.52	3.39	11.3	86.05	4.15	11.3	86.58	4.55	11.3
	RGNN	88.60	5.25	12.1	66.05	2.72	12.1	85.52	8.68	12.1	88.70	3.35	12.1
	HDGCN	89.44	5.37	16.5	68.24	4.05	15.0	86.24	4.86	15.0	92.84	3.43	15.0
	DMGCN	91.31	3.26	7.4	71.95	4.25	6.7	91.56	4.15	6.7	92.54	3.02	6.7
10	DBGCN	84.95	7.67	5.7	63.78	3.77	5.9	91.74	5.38	5.9	95.03	4.18	5.9
	GCB-BLS	88.88	7.53	9.5	69.46	5.66	9.5	92.27	13.94	9.5	96.64	4.12	9.5
	V-IAG	90.97	5.54	12.0	69.53	4.20	12.2	94.17	4.28	12.2	96.72	3.23	12.2
	RGNN	91.97	5.12	13.0	69.65	3.85	13.0	94.96	5.88	13.0	97.94	3.63	13.0
	HDGCN	91.74	4.22	17.7	71.14	2.88	16.2	95.67	3.55	16.2	98.16	2.76	16.2
	DMGCN	94.89	3.93	8.0	72.74	0.82	7.2	95.97	3.68	7.2	98.73	1.15	7.2

*D*: Dimensions of hidden-layer features.

**Table 6:** Effect of hidden-layer number on the model performance. The layer number refers to the number of GCNs in each branch of the model. Moreover, Nikolentzos G. et al. proved the equivalence of one-layer and multilayer GSCs [26]. Therefore, only the layer number of LHC in DMGCN changes.

*L*	Models	SEED			DEAP			DEAP-Arousal			DEAP-Valence		
		ACC	STD	Params	ACC	STD	Params	ACC	STD	Params	ACC	STD	Params
4	DBGCN	73.22	7.06	5.4	58.72	1.11	4.5	82.61	3.34	4.5	84.14	2.91	4.5
	GCB-BLS	76.48	6.24	8.5	62.77	2.34	6.7	89.42	8.15	6.7	90.83	3.94	6.7
	V-IAG	82.83	6.89	11.0	66.26	2.62	9.2	91.89	5.28	9.2	89.54	2.68	9.2
	RGNN	86.7	2.59	11.7	67.71	2.06	9.9	92.65	13.21	9.9	90.59	3.82	9.9
	HDGCN	88.81	4.41	14.4	67.75	2.72	10.3	88.79	3.21	10.3	89.97	2.85	10.3
	DMGCN	90.23	4.29	6.5	69.54	1.18	4.7	85.02	5.25	4.7	92.31	2.55	4.7
6	DBGCN	84.95	7.67	5.9	63.78	3.77	5.0	91.74	5.38	5.0	95.03	4.18	5.0
	GCB-BLS	88.88	7.53	9.5	69.46	5.66	7.7	92.27	13.94	7.7	96.64	4.12	7.7
	V-IAG	90.97	5.54	12.2	69.53	4.2	10.4	94.17	4.28	10.4	96.72	3.23	10.4
	RGNN	91.97	5.12	13.0	69.65	3.85	11.2	94.96	5.88	11.2	97.94	3.63	11.2
	HDGCN	91.74	4.22	16.2	71.14	2.88	12.1	95.67	3.55	12.1	98.16	2.76	12.1
	DMGCN	94.89	3.93	7.2	72.74	0.82	5.4	95.97	3.68	5.4	98.73	1.15	5.4
8	DBGCN	77.44	5.11	6.3	63.52	3.26	5.4	88.36	3.32	5.4	88.72	3.21	5.4
	GCB-BLS	89.38	3.73	10.4	65.72	3.75	8.6	90.75	5.23	8.6	90.91	3.27	8.6
	V-IAG	90.61	7.89	13.5	61.98	3.1	11.7	91.89	1.83	11.7	91.3	6.72	11.7
	RGNN	90.84	8.02	14.2	64.41	8.75	12.4	89.78	3.09	12.4	91.4	7.53	12.4
	HDGCN	92.03	8.66	17.9	69.39	3.23	13.8	93.78	4.19	13.8	93.06	8.27	13.8
	DMGCN	95.07	4.5	8.0	73.97	5.89	6.2	93.59	3.83	6.2	96.73	6.23	6.2
10	DBGCN	72.87	8.28	6.8	59.19	2.99	5.9	79.95	9.02	5.9	80.43	2.77	5.9
	GCB-BLS	86.48	6.19	11.4	60.42	2.62	9.6	86.01	2.74	9.6	82.02	4.03	9.6
	V-IAG	85.83	3.78	14.7	57.95	3.82	12.9	88.00	8.35	12.9	83.42	1.35	12.9
	RGNN	85.22	2.69	15.5	60.78	2.23	13.7	88.36	4.11	13.7	81.77	4.13	13.7
	HDGCN	88.25	4.25	19.7	67.71	2.06	15.6	89.31	1.87	15.6	88.48	1.66	15.6
	DMGCN	94.95	3.12	8.7	72.25	3.47	6.9	94.05	3.03	6.9	95.84	2.85	6.9

*L*: The number of layers of GCNs in each branch of the model.

**Table 7:** Time and space complexity are key metrics for evaluating an algorithm, indicating the worst-case growth in time and the auxiliary space needed. Time complexity impacts the speed of model training and prediction, where high complexity leads to longer times and limits efficient validation and improvement. Space complexity affects the number of parameters, and higher dimensionality requires larger datasets, which is often impractical and increases the risk of overfitting. The units for Bytes and FLOPs are KB and K (one thousand floating point computations per second).

Models	SEED			DEAP			DEAP-Arousal			DEAP-Valence		
	*T*	*S*	*T*/*S*	*T*	*S*	*T*/*S*	*T*	*S*	*T*/*S*	*T*	*S*	*T*/*S*
DBGCN	9.7	4.7	2.1	8.3	4.0	2.1	8.2	4.0	2.1	8.2	4.0	2.1
GCB-BLS	26.6	7.6	3.5	19.9	6.1	3.3	19.6	6.1	3.2	19.6	6.1	3.2
V-IAG	30.7	9.8	3.1	17.3	8.3	2.1	17.2	8.3	2.1	17.2	8.3	2.1
RGNN	29.8	10.4	2.9	19.1	8.9	2.1	19.0	8.9	2.1	19.0	8.9	2.1
HDGCN	26.7	12.9	2.1	20.6	9.6	2.1	20.5	9.6	2.1	20.5	9.6	2.1
DMGCN	36.3	5.8	6.3	34.5	4.3	8.0	32.1	4.3	7.4	34.0	4.3	7.9

*T*: The FLOPs of the model can empirically serve as a proxy for its time complexity, and their unit is kiloflops. *S*: Space complexity can be represented as the GPU memory (in kilobytes) utilized by the model during execution. T/S: The ratio between FLOPs and memory usage (in kilobytes) can be interpreted as a measure of computational density.

**Table 9:** Summary of models in ablation experiments. DMGCN-I: representations from HDGIL are directly used for classification through 2-layer MLP (multilayer perceptron), instead of M2FC (multi-level feature fusion classifier); DMGCN-II: GCN (graph convolution network) is replaced with GSC (global subgraph checker); DMGCN-III: GCN is replaced with LHC (local hierarchy checker), and the combination of the concatenation operation, Euclidean mean pooling and 2-layer MLP are regarded as a GIL (geometry interactive layer); DMGCN-IV: similarly to in DMGCN-III, the combination of the concatenation operation, Euclidean mean pooling and 2-layer MLP are regarded as a GIL.

Method	M2FC	GSC	LHC	GIL
DMGCN	✓	✓	✓	✓
DMGCN-I	✗	✓	✓	✓
DMGCN-II	✓	✗	✓	✓
DMGCN-III	✓	✓	✗	✓
DMGCN-IV	✓	✓	✓	✗

**Table 10:** Effect of hidden-layer dimension on DMGCN’s performance. The error ratio represents the proportion of samples requiring more than 20 iterations out of the total samples. The maximum number of iterations was set to 20. The time was 1 h.

Dimension	SEED				DEAP				DEAP-Arousal				DEAP-Valence			
	ACC	STD	*T*	ρ	ACC	STD	*T*	ρ	ACC	STD	*T*	ρ	ACC	STD	*T*	ρ
5	90.12	13.22	0.36	1.40	65.94	1.41	0.37	2.11	82.40	6.46	0.32	2.16	87.22	7.71	0.33	1.63
10	94.89	3.93	0.55	10.75	72.74	0.82	0.53	13.12	95.97	3.68	0.51	14.42	98.73	1.15	0.53	11.49
15	94.28	10.96	0.67	37.22	68.50	6.94	0.64	37.80	89.79	10.94	0.61	38.30	96.36	2.06	0.65	19.05
20	82.29	12.28	0.86	54.54	56.15	14.01	0.87	82.60	80.87	7.57	0.85	61.70	89.95	9.02	0.82	56.29
30	64.88	6.87	0.97	96.52	50.69	5.79	0.93	97.25	70.84	17.53	0.96	87.99	74.96	8.94	0.93	85.49

*T* is the time, in hours, spent on training the model per epoch. ρ: The ratio of samples failing to converge to the total number of samples within the maximum number of iterations.

**Table 11:** Effect of numerical precision on DMGCN’s performance. The unit of the absolute error and relative error is percentage. The absolute error $\left(10^{-3}∼10^{-5}\right)$ and relative error (1∼0.01%) are formulated as $|X_{t}-X_{t-1}|$ and $\left|\frac{X_{t}-X_{t-1}}{X_{t}}\right|$, respectively. The iteration can be terminated when the absolute or relative error is less than the corresponding threshold.

ϵ	ε	SEED				DEAP				DEAP-Arousal				DEAP-Valence			
		ACC	STD	*T*	ρ	ACC	STD	*T*	ρ	ACC	STD	*T*	ρ	ACC	STD	*T*	ρ
$10^{-3}$	1	77.50	8.10	0.45	0.04	60.84	7.57	0.48	0.02	76.63	6.99	0.45	0.02	71.64	6.87	0.46	0.06
	0.1	80.69	5.79	0.57	8.81	65.30	6.72	0.52	13.11	84.96	8.94	0.56	2.85	87.50	8.10	0.49	3.50
	0.01	80.30	6.73	0.68	26.70	56.63	6.99	0.58	28.50	81.64	3.57	0.55	18.82	84.75	4.24	0.56	25.38
$10^{-4}$	1	79.35	6.98	0.45	0.03	60.40	8.13	0.46	0.09	77.32	5.94	0.42	0.07	76.30	6.91	0.45	0.06
	0.1	94.89	3.93	0.55	10.75	72.74	0.82	0.53	13.12	95.97	3.68	0.51	14.42	98.73	1.15	0.53	11.49
	0.01	70.37	7.68	0.63	21.63	66.00	3.92	0.54	31.05	86.22	11.52	0.66	29.53	82.54	3.97	0.60	30.56
$10^{-5}$	1	79.70	8.03	0.55	0.03	60.81	5.79	0.49	0.09	81.04	6.28	0.49	0.05	71.04	6.35	0.46	0.06
	0.1	94.55	5.25	0.58	34.31	72.41	5.73	0.63	46.31	89.85	3.08	0.68	31.91	85.33	5.43	0.60	26.92
	0.01	71.93	4.92	0.76	64.91	65.58	4.24	0.71	79.04	78.57	4.24	0.71	68.16	82.56	3.22	0.71	74.48

ϵ: Absolute error. ε: Relative error. ρ: The ratio of samples failing to converge to the total number of samples within the maximum number of iterations.

## Data Availability

The database used in this study is publicly available at the following websites: DEAP—http://www.eecs.qmul.ac.uk/mmv/datasets/deap/ (accessed on 6 June 2024); SEED—https://bcmi.sjtu.edu.cn/home/seed/seed.html (accessed on 6 June 2024).
